# Stabilized COre gene and Pathway Election uncovers pan-cancer shared pathways and a cancer-specific driver

**DOI:** 10.1126/sciadv.abo2846

**Published:** 2022-12-21

**Authors:** Pathum Kossinna, Weijia Cai, Xuewen Lu, Carrie S. Shemanko, Qingrun Zhang

**Affiliations:** ^1^ Department of Biochemistry & Molecular Biology, University of Calgary, Calgary, Alberta T2N 1N4, Canada.; ^2^ Alberta Children’s Hospital Research Institute, University of Calgary, Calgary, Alberta T2N 1N4, Canada.; ^3^ Department of Cancer Biology, Sidney Kimmel Cancer Center, Thomas Jefferson University, Philadelphia, PA 19107, USA.; ^4^ Department of Mathematics and Statistics, University of Calgary, Calgary, Alberta T2N 1N4, Canada.; ^5^ Department of Biological Sciences, University of Calgary, Calgary, Alberta T2N 1N4, Canada.; ^6^ Arnie Charbonneau Cancer Research Institute, University of Calgary, Calgary, Alberta T2N 1N4, Canada.

## Abstract

Approaches systematically characterizing interactions via transcriptomic data usually follow two systems: (i) coexpression network analyses focusing on correlations between genes and (ii) linear regressions (usually regularized) to select multiple genes jointly. Both suffer from the problem of stability: A slight change of parameterization or dataset could lead to marked alterations of outcomes. Here, we propose Stabilized COre gene and Pathway Election (SCOPE), a tool integrating bootstrapped least absolute shrinkage and selection operator and coexpression analysis, leading to robust outcomes insensitive to variations in data. By applying SCOPE to six cancer expression datasets (BRCA, COAD, KIRC, LUAD, PRAD, and THCA) in The Cancer Genome Atlas, we identified core genes capturing interaction effects in crucial pan-cancer pathways related to genome instability and DNA damage response. Moreover, we highlighted the pivotal role of *CD63* as an oncogenic driver and a potential therapeutic target in kidney cancer. SCOPE enables stabilized investigations toward complex interactions using transcriptome data.

## Teaser

A statistical model identifies core genes and pathways from transcriptome data robust to changes of data.

## INTRODUCTION

Understanding the process of pathogenesis and discovering previously unidentified therapeutic targets require discovery of the underlying driver genes in relevant pathways (*1*–*3*). However, determination of the “driver” role of a gene through experimental investigation has only been possible for a handful of genes because of the time-consuming and expensive nature of such experiments. Thus, in silico analysis to narrow down candidates of potential genes is vital. Current methods of identifying driver genes involve multiomics data (*4*) and often use known biological pathways (*5*). Among multiomics data, transcriptomes, i.e., gene expression data, play a pivotal role in biological processes and are the most available form of omics data for many diseases including cancers. As such, analyzing transcriptomic data is usually the first step in omics-directed characterization of diseases.

In practice, selecting differentially expressed (DE) genes by contrasting expression levels in disease and control tissues has been broadly used for the exploration of biological mechanisms of various diseases. Largely because of its simplicity, single-gene-based DE analysis is the most popular method adapted by many researchers (*6*). From the perspective of systems biology, it is natural to expect that advanced models analyzing multiple genes jointly should lead to additional in-depth understanding of disease pathology.

Unfortunately, instability of such complex models involving multiple genes appears to be a serious problem; in many situations, current methods do not generate consistent results. For instance, in a typical coexpression network–based analysis, a gene network is built with its nodes representing genes and edges based on their coexpression. The genes that are highly connected with other genes in the network, called “hub” genes, are expected to be important in pathology (*7*). As such, many pipelines discovering driver genes incorporate information from coexpression networks and these hub genes into the next phase of multiomics approaches (*8*–*10*). It has, however, been noted that hub genes are not stable, and they are not guaranteed to be driver genes (*11*).

Regularized multiple regression methods, which optimize an objective function by adding a regularization term to a likelihood, are widely used in many domains (*12*–*14*) including biomarker selection using transcriptomic data. LASSO (least absolute shrinkage and selection operator) and ridge regression are two representative methods of this nature (*15*, *16*). The choice of regularization plays a notable role in the information supplied by the final model: Ridge regression will lead to a model containing a large number of genes (*15*), which may confer a high predictive power at the cost of little meaningful information for functional characterization; LASSO, in contrast, retains fewer genes (*16*) but is inherently unstable in the presence of highly correlated variables (*17*, *18*), which is unfortunately the case of transcriptome data. While a logistic LASSO regression can usually identify significant variables in determining case and control, it also tends to provide completely different outcomes with different parameterizations or, even by running a similarly parameterized model multiple times over, slightly different data (*17*, *19*). That is why, historically, as far as we understand, there have been few efforts using such feature selection methods in identifying underlying genes from transcriptomic data.

Coexpression network analyses and regularized multiple regressions form disconnected fields, which are by themselves unable to produce stable results offering insights into disease pathology. We propose the Stabilized COre gene and Pathway Election (SCOPE), a new tool to reliably discover candidate genes and pathways using transcriptomic data. The new framework represents a synergy between coexpression network analysis and regularized multiple regressions, with two layers of stabilization integrated.

To assess the theoretical properties of SCOPE, we first conducted a simulation study where various scenarios of signal-to-noise ratio, nonlinearity, interaction, and coexpression structures are considered and evaluated. The results showed SCOPE’s advantage in most configurations over state-of-the-art regularization methods.

As a proof of concept in real data, we applied SCOPE to six cancer datasets from The Cancer Genome Atlas (TCGA) (*20*) [breast invasive carcinoma (BRCA), colon adenocarcinoma (COAD), kidney renal clear cell carcinoma (KIRC), lung adenocarcinoma (LUAD), prostate adenocarcinoma (PRAD), and thyroid carcinoma (THCA) with 1,041-111, 480-70, 483-54, 387-37, 458-50, and 444-53 “primary tumor”–“normal tissue” samples, respectively] to identify novel core genes and their related pathways. Thorough comparisons were carried out against standard methods including LASSO (for the selection step only) and differential expression (DE) analysis as well as differential coexpression (DiffCoEx) analysis. As expected, the core genes selected by SCOPE-Stabilized LASSO are stable with respect to small changes of the input datasets. Despite being significantly fewer than the typical set of genes identified by a standard LASSO, the SCOPE-identified core genes remain highly predictive. Notably, as another line of evidence at the pathway level, pathways identified by SCOPE show significant within- and pan-cancer overlap. In contrast, standard DiffCoEx analysis led to significantly lower overlaps across and within cancers. Moreover, as a confirmation by using connectivity analysis, we found that the core genes play central roles in the shared pathways.

To discover previously unknown insights into cancer pathology based on the stably identified core genes and pathways, we have carefully annotated the data based on our insights into cancers and from the literature. Notably, we shed light on the critical role of *CD63* in the Von Hippel–Lindau tumor suppressor (*VHL*)–hypoxia-inducible factor 1 subunit α (*HIF1A*)/hypoxia-inducible factor 2α (*HIF2A*)–vascular endothelial growth factor A (*VEGFA*) protein (*VHL*-*HIF*-*VEGF*/*VEGFR*) axis (*21*), a putative key driver that governs tumorigenesis in kidney cancer. These discoveries may provide interesting insights into the mechanism of cancer, identifying concrete targets for further experimental follow-ups.

## RESULTS

### The SCOPE framework

**Fig. 1.**
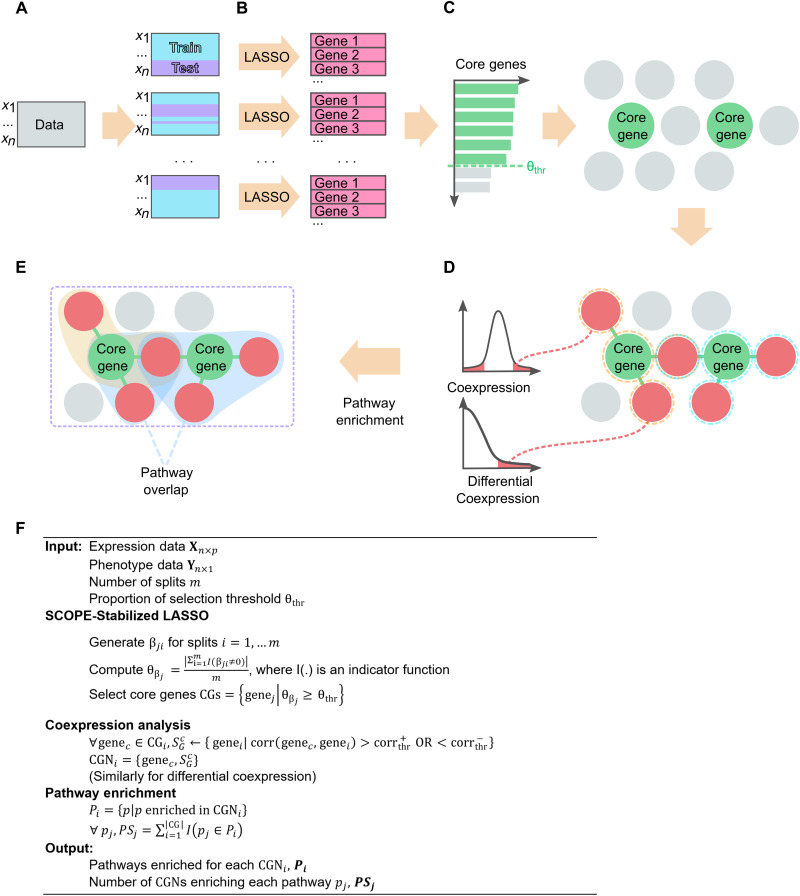
Overview of SCOPE framework. (**A**) Expression data are split multiple times randomly into typical training/test splits with a consistent phenotype ratio as in the original data. (**B**) LASSO models are trained on each of the splits, and the genes selected in each split are recorded. (**C**) Selected genes are ordered by the frequency of occurrence in the splits. On the basis of a cutoff θ_thr_ (dashed green line), core genes are identified and used to identify the CGNs in (D). (**D**) CGNs are identified, indicated by genes circled in orange and blue dashed lines. Null distributions of both DiffCoEx and coexpression are used to identify genes significantly interacting with the identified core genes. (**E**) Pathway enrichment analysis is conducted for each CGN, and the overlap between CGN-directed pathways will be identified. Last, substantially overlapped pathways and core genes will be the output. (**F**) The algorithm in a simplified high-level pseudo-code. The full version of the algorithm is presented in the Supplementary Materials.

SCOPE begins by conducting multiple regression by using a stabilized extension of LASSO, here termed SCOPE-Stabilized LASSO, which uses a bootstrap of multiple LASSO models (Fig. 1, A and B), leading to a handful of core genes robust to statistical instability (Fig. 1C). These core genes differ from hub genes in that they are not identified because of their interconnectedness but because of their power in prediction while still being stable across random samples. These core genes are then used as seed genes for further coexpression and DiffCoEx analysis (Fig. 1D), constructing core gene networks (CGNs). These CGNs are then piped into pathway enrichment analysis (Fig. 1E). The pathways learned from each CGN are lastly intersected to provide another level of stabilization (Fig. 1E). A high-level pseudo-code is included in Fig. 1F, and the detailed algorithms and design considerations are provided in Materials and Methods and the Supplementary Materials. This framework incorporates both optimizations brought by multiple regressions and gene-gene interactions identified by coexpression analysis while retaining stability in large part due to two levels of stabilization.

### SCOPE’s outcome is robust to its most key tuning parameters

The SCOPE framework uses several parameters that may be tuned to produce biologically relevant results. θ_thr_ ( ∈ [0,1]) determines the number of core genes identified by SCOPE, with higher values reducing the number of core genes selected. *r*_thr_ ( ∈ [0,1]) and $r_{\mathrm{thr}}^{D}\;(\in [0,1])$, which are the coexpression and DiffCoEx percentile thresholds, respectively, are used to determine the cutoff of significance of secondary genes used to construct CGNs. The number of iterations *n*_iter_(∈ $Z^{+}$) and the sample split proportion *s*_prop_ ( ∈ [0,1]) are the parameters relevant to the SCOPE-Stabilized LASSO step of the framework.The outcome of SCOPE is highly robust to reasonable changes in its parameters’ values. Figure S1 gives a visual indication of the influence of these parameters on the overall pathway overlap score (POS; a measure of the scale of shared pathway enrichment across multiple datasets ranging from 0 to the number of datasets) across the six cancers studied in the TCGA database. Evidently, the maximum POS remains robust throughout changes in all parameters (fig. S1, A to D), except for θ_thr_ where stringent values lower the POS lightly but with an observable trend (fig. S1E). Thus, θ_thr_ may be tuned on the basis of the situation and upon observing the frequency distribution of the selected genes and upon the feasibility of experimental follow-up.

### Simulation study unveils the performance of SCOPE in identifying core genes and related pathways in simulations

**Fig. 2.**
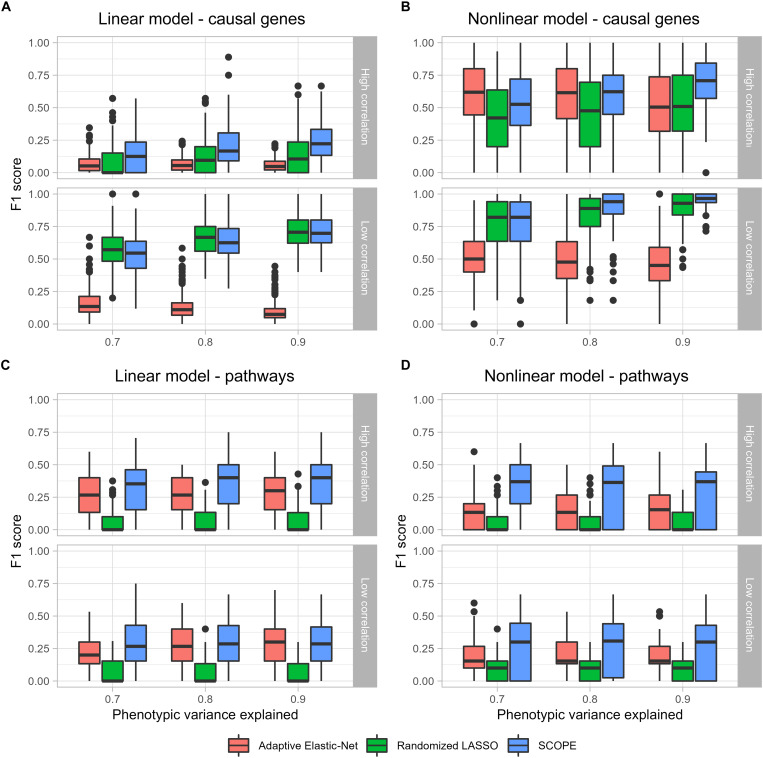
Simulations comparing the performance of SCOPE, Adaptive Elastic-Net, and randomized LASSO. F1 score {= TP/[TP + 0.5 × (FP+ FN)]} calculated for the accuracy of Adaptive Elastic-Net, randomized LASSO, and SCOPE models in identifying causal genes and pathways simulated in the gene expression data with 670 samples. (**A**) and (**B**) indicate the ability of SCOPE to identify causal genes with better accuracy, particularly in scenarios with higher correlations of the core genes in both linear and nonlinear phenotypes, respectively. (**C**) and (**D**) demonstrate the ability of SCOPE to identify core pathways among the top 10 pathways enriched using each method with higher accuracy. Note that in the nonlinear model, we assumed that the genes participating in interactions are known as a priori; otherwise, the powers of all three methods are close to zero. Please see detailed justifications in Materials and Methods.

The main simulations were conducted using 670 samples of whole-blood tissue expression from the Genotype-Tissue Expression (GTEx) Consortium (*22*) to compare the performance of SCOPE, Adaptive Elastic-Net (*23*), and randomized LASSO (*17*). Overall, simulations uncovered SCOPE’s distinct competitive advantage over other methods in discovering core genes and their related pathways. Simulations were conducted under a variety of scenarios simulating noise-to-signal ratios, linear and nonlinear phenotypes, and correlation structures, which are detailed further in Materials and Methods. In each simulation, we first set up “gold-standard” core pathways and then selected core genes related to these pathways that are either (i) highly or (ii) lowly correlated with these pathways (Materials and Methods). These core genes and a few randomly selected additional genes were then deemed as “causal genes,” which were used to generate a binary phenotype through both linear and nonlinear models consisting of interactions. The three methods are then evaluated on their ability to identify both causal genes and core pathways in terms of F1 scores. We present the results using two different cutoffs: The first is based on the top 10 pathways that may reflect the practice of looking at the top pathways for experimental validations (Fig. 2 with a breakdown of prediction metrics provided in tables S1 and S2); the second is based on pathways identified with false discovery rate (FDR) < 0.05 that may be statistically rigorous (fig. S2 with a further breakdown in table S3). Since the above comparisons focus on the performance measured only by the final outcome (causal genes and core pathways), to characterize the contribution of multiple steps, we also analyzed the ability in identifying core genes, the intermediate outcome (fig. S3 with a further breakdown in table S4). Simulations were also run on smaller random subsets of the data as 500 samples (figs. S4 to S6 and tables S5 to S8) and 250 samples (figs. S7 to S9 and tables S9 to S12).

Under both the linear and nonlinear models, SCOPE-Stabilized LASSO was able to consistently perform competitively with the other methods and had a distinct advantage in identifying causal genes in the presence of highly correlated core genes (Fig. 2, A and B). By inspecting the corresponding performance for core genes (fig. S3), especially in the case of highly correlated core genes, one can see that the performance of all models is lowered. Evidently, SCOPE can better identify highly correlated core genes in contrast to other methods, which gain power more through the discovery of causal genes that are not core genes. While randomized LASSO performs similarly to SCOPE-Stabilized LASSO in the low-correlation scenario and the Adaptive Elastic-Net performs relatively well in the presence of a nonlinear phenotype with highly correlated core genes, SCOPE-Stabilized LASSO performs the best, on average, across all scenarios. In real data analysis, one is unaware of the level of correlations and the linearity of the phenotype; therefore, SCOPE-Stabilized LASSO would be the best tool of choice.

Pathway enrichment revealed that SCOPE was able to better identify core pathways (Fig. 2, C and D) when considering the top pathways enriched. In this scenario, randomized LASSO performs the poorest because of the lower number of genes identified in comparison to the Adaptive Elastic-Net and SCOPE. However, the larger number of genes identified by Adaptive Elastic-Net, which led to the larger number of false-positive causal genes (thus a lower F1 score in identifying the same), enabled Adaptive Elastic-Net to achieve a similar performance to SCOPE in the linear model (Fig. 2C) but still provided an edge to the more comprehensive coexpression network analysis of SCOPE in the nonlinear scenario (Fig. 2D).

### SCOPE stably selected considerably fewer core genes while retaining predictive power

**Fig. 3.**
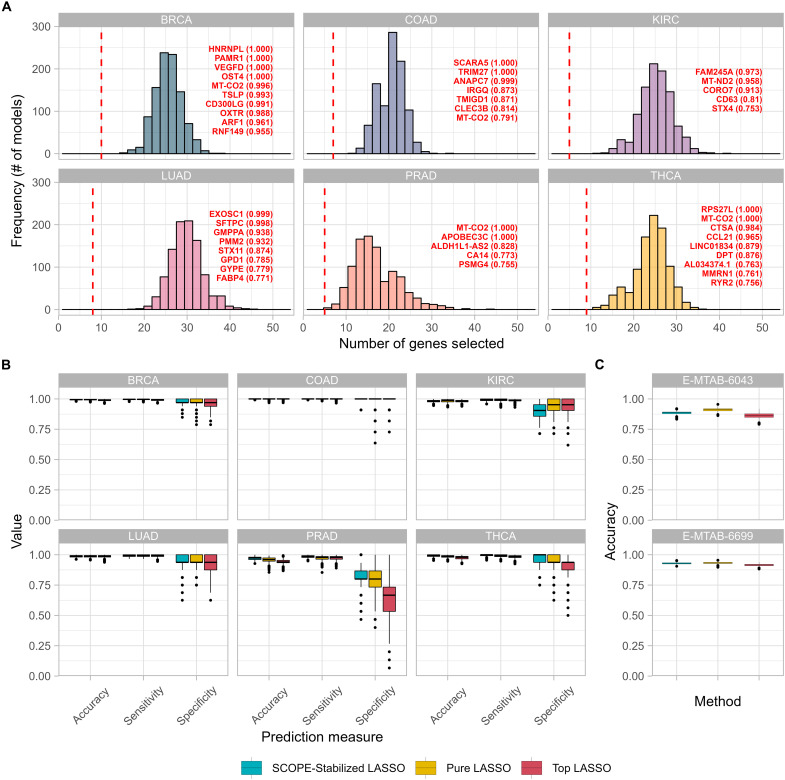
Comparison of SCOPE to standard LASSO in stability and predictive accuracy. (**A**) Histograms of the number of genes selected by standard LASSO (colored distributions) in comparison to SCOPE (vertical red dashed lines) for each cancer. The thresholds chosen for SCOPE-selected core genes were varied: θ_thr_ = 0.90 for BRCA and θ_thr_ = 0.75 for KIRC, LUAD, COAD, PRAD, and THCA. These thresholds resulted in 5 to 10 core genes being identified per cancer, identified in the six panels for each cancer. (**B**) Prediction metrics for SCOPE (core genes) in comparison to standard LASSO in terms of accuracy = [True Positives (TP) + True Negatives (TN)]/[TP + TN + False Positives (FP) + False Negatives (FN)], sensitivity, and specificity. (**C**) Prediction accuracy = (TP + TN)/(TP + TN + FP + FN) for two independent microarray datasets for lung cancer was obtained. In the case of SCOPE, the same core genes identified and indicated in table S1 were used. For standard LASSO, multiple sets of genes selected by independent LASSO runs in the TCGA LUAD dataset were used to assess the varied distribution (due to instability), and for top LASSO, the top genes in each standard LASSO equal in number to those selected by SCOPE-Stabilized LASSO were used. Prediction measures are calculated on the basis of the true labels of the data (tumor/normal) and the predicted labels on the test data sampled from the original TCGA data.

SCOPE-Stabilized LASSO identified significantly fewer genes among different splits of the data (Fig. 3A, vertical red dashed lines). The consistently selected genes are named in Fig. 3A with details in table S13. In contrast, on the same data, genes selected by a standard LASSO are much more numerous and vary widely from around 10 to 45 genes (Fig. 3A, colored distributions), documenting the instability of gene selection by standard LASSO.

Despite being much smaller in number, the predictive power (in predicting normal/tumor phenotypes) of SCOPE-selected core genes is close to that obtained by standard LASSO. In-sample validation shows that the few genes identified by SCOPE confer almost the same, and sometimes even higher, predictive power compared with the many genes selected by standard LASSO (Fig. 3B). Restricting the LASSO to use the top (indicated by the highest absolute coefficients) genes, equal in number to the number of genes used by SCOPE, reveals poorer predictive power in comparison with both the standard LASSO and SCOPE-Stabilized LASSO.

We also resorted to external data validation using two microarray datasets (*24*, *25*) based on the set of core genes identified by SCOPE-Stabilized LASSO (listed in table S13) and the multiple runs of standard LASSO (mirroring in practice the range that different people might achieve on the basis of the genes that they ended up identifying) as well as the top genes identified in each of the standard LASSO runs. Evidently, the predictive accuracy remains close to one using standard LASSO (Fig. 3C) and higher in accuracy than using the top genes identified by any single LASSO model. Internal and external validation highlighted the ability of SCOPE-Stabilized LASSO to identify a highly predictive handful of genes that are comparable in prediction accuracy to the many-fold larger number of genes selected by a standard LASSO model. The small margin also indicates that, while models including more genes may be slightly more predictive, they may not all be vital to tumorigenesis. Furthermore, such a large number of genes could be extremely costly to experimentally validate and thus are not an ideal outcome of in silico methods, a problem relieved by SCOPE-Stabilized LASSO selection.

The consistency and stability of SCOPE-Stabilized LASSO over standard LASSO were demonstrated by looking at the replicability over multiple runs on the same data (with different splits of training/testing samples). The comparison was conducted by randomly splitting the data into training and testing samples 100 times, using a different random seed for each split. This reflects the effect of choosing a different training sample in a typical usage scenario. Proportions of runs in which genes were selected are shown in table S14, illustrating the high level of stability obtained by SCOPE-Stabilized LASSO over standard LASSO.

### SCOPE identified pan-cancer pathways, focusing on DNA replication and repair

Via standard coexpression analysis of gene networks, the core genes selected by the SCOPE-Stabilized LASSO were used to form their corresponding CGNs, which, in turn, were used to identify pathways based on pathway enrichment analysis (Materials and Methods). The pathways that are identified by multiple CGNs are the output of SCOPE (table S15). Many of these pathways fall into the categories of “cell growth and death,” “replication and repair,” and “folding, sorting, and degradation.” These pathways are highly related to cancer cell immortality and cancer genome damage response. A similar protocol was also conducted using DiffCoEx analysis by looking at pathways identified by multiple modules (table S16).

To further assess the stability of SCOPE, we analyzed the sharing of core genes between cancers. At the gene level, *MT-CO2* was identified as a core gene by SCOPE in four of the six cancers. This gene produces the cytochrome c oxidase subunit 2 protein, which is essential in a mitochondrial process associated with oxidative phosphorylation. Besides *MT-CO2*, no other core gene is shared among cancers, indicating that different cancers may have different core genes if one does not look at higher levels such as pathways.

**Fig. 4.**
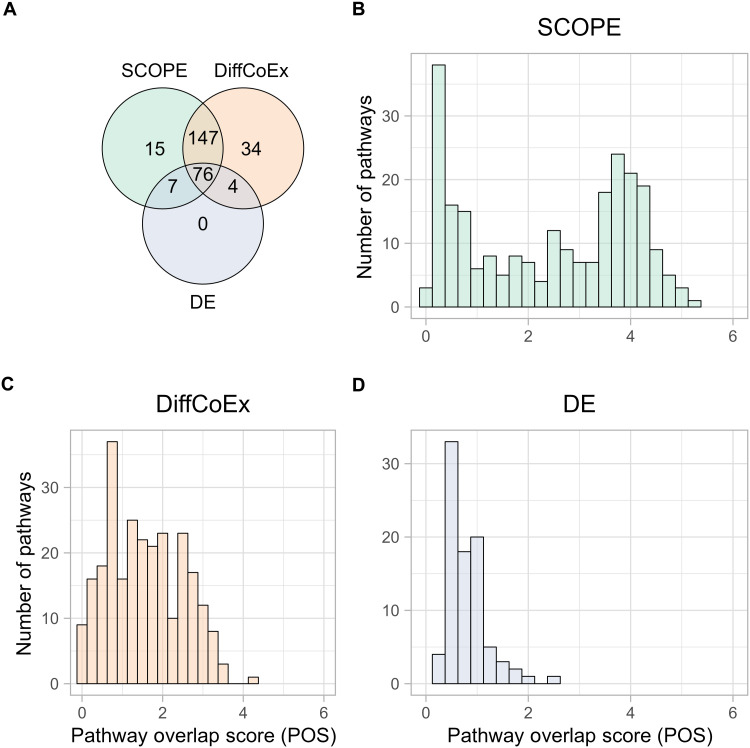
Comparison of SCOPE to alternative methods on pathway identifications. (**A**) Pathways identified by DiffCoEx, DE, and SCOPE are compared for uniqueness and sharing. (**B** to **D**) POS, which indicates the level of enrichment of a pathway across multiple cancers, is contrasted among the three methods. (B) SCOPE uncovers both cancer-specific (notable by the spike in lower POS) and pan-cancer shared pathways (notable by the spike in higher POS), while both DiffCoEx (C) and DE (D) appear to be more cancer specific than SCOPE as evidenced by the lower distribution of POS.

We then characterized the sharing of pathways across cancers. To quantify the extent of sharing, we first formed a within-cancer statistic, π_cancer_, which denotes the proportion of genes contained in CGNs out of the total number of genes in each pathway. Then, the POS was calculated as the summation of the π_cancer_ values over all six cancers. Higher values intuitively indicate higher overlap of the pathway across cancers. Contrasting the results of the three tools, namely, SCOPE, DE, and DiffCoEx, despite their substantial sharing in terms of pathways identified (Fig. 4A), showed quite different landscapes in terms of sharing between cancers. Evidently, SCOPE identified both cancer-specific and pan-cancer pathways characterized by its two-spike distribution of POS: The cluster at the low-POS end stands for cancer specific, while the cluster at the high-POS end indicates pan-caner pathways (Fig. 4B). In contrast, both DE and DiffCoEx distributions have only one spike at the low-POS spectrum (Fig. 4, C and D). These POS distributions are further detailed in table S17 for SCOPE, DiffCoEx, and DE, evidencing the potential drawback of DiffCoEx and DE in their inability to repetitively identify key pathways that could be universally vital in cancers. This distinction between SCOPE and standard methods suggests that SCOPE’s stability in discovering pathways can reveal key pathways even in different cancers.

**Fig. 5.**
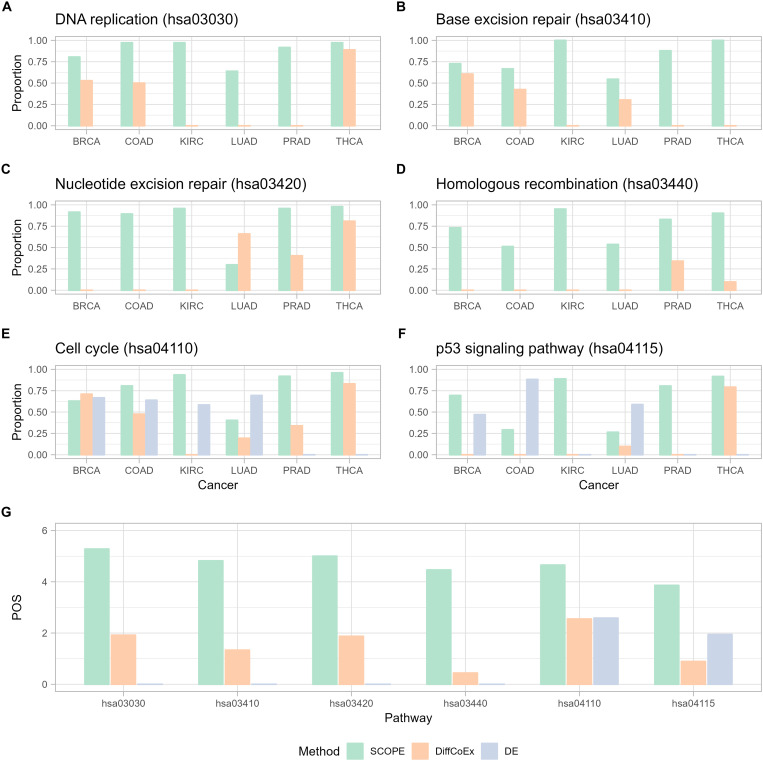
Comparison of proportion of genes in each cancer (π) identified by SCOPE in contrast to DiffCoEx and DE in pathways related to hallmarks of cancers. Pathways shown are (**A**) DNA replication (has03030), (**B**) base excision repair (hsa03410), (**C**) nucleotide excision repair (hsa03420), (**D**) homologous recombination (hsa03440), (**E**) cell cycle (hsa04110), and (**F**) p53 signaling pathway (hsa04115). (**G**) Comparison of POS across the three methods for the same pathways (A to G). POS is calculated as the sum of π values across the cancers for each pathway. Higher values indicate higher discovery of genes related to each pathway across cancers.

We then annotated pathways that were identified by SCOPE to check whether they were relevant. Investigating pathways related to the hallmarks of cancer (*26*) and the proportion of genes in each of these pathways by each of the three methods (Fig. 5, A to F) reveals that SCOPE identifies these hallmarks across cancers quite significantly. Figure 5G looks at the POS for these hallmark pathways across the different cancers. SCOPE stands out by identifying the highest proportion of genes involved in these pathways.

By analyzing the literature further, we realized that the pan-cancer pathways revealed by SCOPE are biologically meaningful. Overlapped pathways enriched using overrepresentation analysis on the Kyoto Encyclopedia of Genes and Genomes (KEGG) database, excluding any pathways that had no enrichment for one or more cancers, immediately reveal the high enrichment of pathways related to regulating the universal level of DNA/RNA/protein and, notably, the pathways related to DNA repair. The most notable characteristic of cancer is the unlimited growth of cancer cells, which also links to the cell cycle pathway (*26*). Mechanistically, they need readily available supplies of materials for cell growth and replication, e.g., more DNA replication, more RNAs transcribed by RNA polymerase and spliced by the spliceosome, and more proteins translated by ribosome. The increased supplies are also likely due to less RNA degradation, less protein degradation by the proteasome, and more N-glycan biosynthesis for N-linked glycosylation, one of the most abundant protein modifications that play a critical role in tumorigenesis (*27*). In addition, increased DNA replication accumulates errors as DNA mutations. Mutations inactivating tumor suppressor genes can further accelerate the accumulation of mutations, partially through defective DNA damage repair, and result in genome instability, a hallmark of all cancers (*26*). Hence, this result demonstrates that the core genes identified by SCOPE-Stabilized LASSO are stably connected with pathways essential to tumor growth and/or associated with the fundamental hallmarks of any type of cancer.

### Pan-cancer pathways exhibit contrastive interaction patterns centered by core genes

**Fig. 6.**
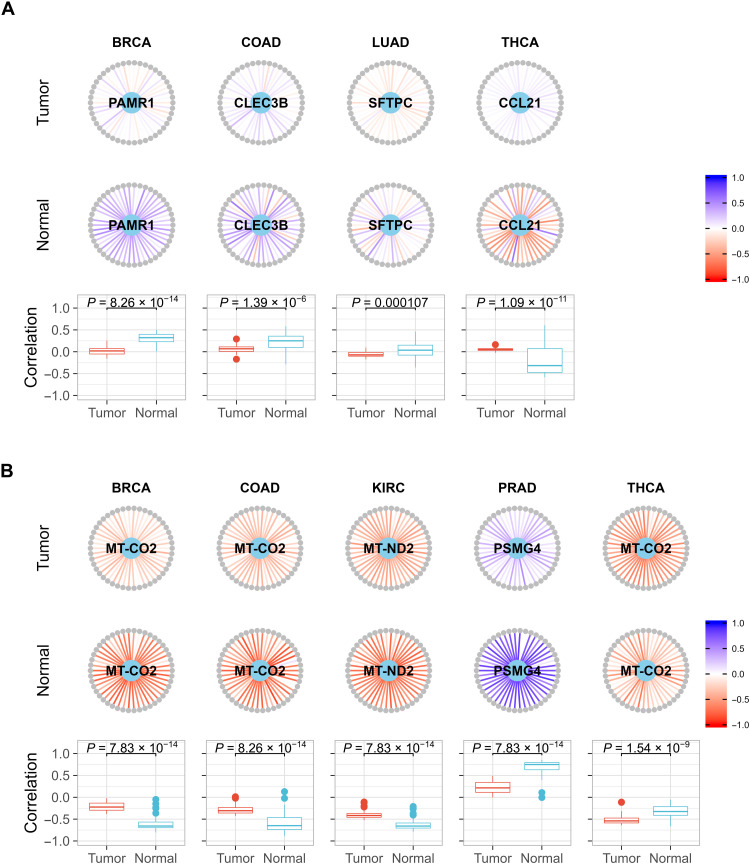
Example of the roles of core genes in a pan-cancer pathway uncovered by SCOPE. The nucleotide excision repair pathway (hsa03420) is used in this example. Core genes (light blue) are in the center of the network with the genes in this pathway (gray) arranged in a circle. Pearson’s correlation coefficients are indicated as edges ranging from −1 (red) to +1 (blue). The names of the genes and their correlations with the core genes are noted in table S18. Boxplots contrast the distributions of two sets of correlations (tumor versus normal) along with the *P* value for the Kolmogorov-Smirnov test, with the null hypothesis being that the two samples were drawn from the same distribution. (**A**) Core genes are DE, and (**B**) core genes are not DE.

To further confirm the roles of the core genes in their discovered pathways, we calculated the correlations between a core gene and all the genes in the corresponding pathway. The core genes exhibit highly disruptive patterns in the coexpression network. Taking the nucleotide excision repair pathway network across multiple cancers as an example, the coexpression networks fundamentally differ in structure and intensity with respect to the core genes (Fig. 6, A and B). Despite different cancers using different core genes, the correlations between the core genes and the other genes in the same pathway are universally higher or lower in the tumor tissue. Many of the core genes are not DE (Fig. 6B), indicating that core genes may contribute to cancers by disrupting their interactions, although their own expression levels are not significantly altered. These results further strengthen the role that core genes appear to play in the pathology of these cancers.

Other coexpression networks centralized by other core genes are provided in figs. S10 to S14 and further detailed in table S18, showing switched (opposing) correlation patterns (and, in some cases, an absence of correlations) when contrasting tumor tissue to normal tissue. These switched correlations appear to indicate that the core genes identified by the SCOPE-Stabilized LASSO method are highly connected genes that are indicators of the proper functioning of these pathways, if not responsible for mediating these pathways.

In addition to the above pan-cancer shared pathway analysis, SCOPE also identified cancer-specific pathways, some of which show contrastive connectivity patterns. For instance, in breast cancer, glyoxylate and dicarboxylate metabolism, fatty acid degradation, and regulation of lipolysis in adipocytes were found (fig. S15, A to C) (*28*). For colon cancer, bile secretion, mineral absorption, and proximal tubule bicarbonate reclamation were highlighted (fig. S15, D to F). Out of the SCOPE-identified colon cancer–specific pathways, 90% are classified as being in the category of metabolism, while other cancers do not show such patterns.

### In-depth annotation reveals hypothetical CD63-centered mechanism in kidney cancer

**Fig. 7.**
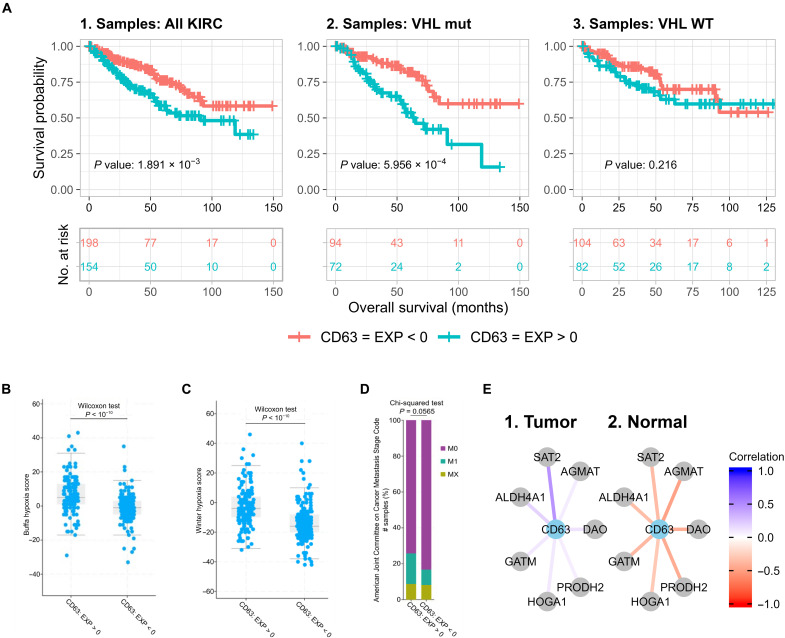
Hypothetical role of *CD63* in kidney cancer. EXP < 0 indicates samples in which the expression level of the gene *CD63* is lower than the arithmetic mean of the expression levels of the gene across all samples, while EXP > 0 indicates a value higher than mean expression levels. (**A**) Survival plots of patients considering differing expression of *CD63* in (1) all patients in the KIRC dataset, (2) patients with *VHL* mutation, and (3) patients with *VHL* wild type (WT). (**B**) and (**C**) indicate that a higher expression of *CD63* correlates with a higher expression of hypoxia-related genes profiled by two (*73*, *74*) well-known hypoxia gene signatures, while (**D**) indicates the relationship between *CD63* and metastasis in kidney cancers. (**E**) (1 and 2) Connectivity network suggesting the role that *CD63* plays in the arginine and proline metabolism pathway with key genes involved in the pathway [data and figures of (A) to (E) are derived from the cBioPortal website (www.cbioportal.org/)].

Among the five core genes selected by SCOPE in kidney cancer (KIRC), *CD63* plays an indispensable role in *VEGFR2* activation in response to *VEGF* (*29*). Notably, aberrant activation of the *VEGF*-*VEGFR* axis is a pivotal driver in kidney cancer since more than 60% of patients with kidney cancer harbor *VHL* mutations (*30*). Inactivated *VHL* fails to degrade HIF α subunits (*HIF*α) in kidney cancer cells. The accumulation of HIFα induces the transcription of hypoxia-related genes and activation of hypoxia signaling in the presence of oxygen. As a key downstream target of *HIF*α, *VEGF* expression and secretion further cause autocrine or paracrine activation of the VEGFR signaling pathway (*21*). Hence, *CD63* probably plays an oncogenic role in kidney cancer. Consistently, high mRNA level of *CD63* associates with adverse prognosis in patients with KIRC (*P* = 0.0019; Fig. 7A, 1). In contrast, there is no such relationship in the other five cancer types (fig. S16). In agreement with *CD63*’s role in the activation of *VEGFR* signaling pathway, which is driven by *VHL* mutations in KIRC, the association is more significant in *VHL*-mutated cohorts (*P* = 0.0006; Fig. 7A, 2) than in *VHL*–wild-type cohorts (*P* = 0.216; Fig. 7A, 3). Along this line, high expression of *CD63* in kidney tumors correlates with a hypoxia gene signature assessed by two different scores (Fig. 7, B and C). *CD63* is also known as a marker of exosomes, extracellular vesicles secreted by cells (*31*). In agreement with the fact that exosomes can contribute to metastasis (*32*), *CD63* shows a tendency to be correlated with metastasis in patients with KIRC although barely above the significant cutoff of 0.05 (*P* = 0.0565; Fig. 7D). In particular, *CD63* knockout mice are viable, fertile, and almost normal except for an altered water balance, such as increased urinary flow, water intake, reduced urine osmolality, and a higher fecal water content (*33*). This does not only suggest that *CD63* plays a critical and specific role in kidney pathology, and consequently in kidney tumorigenesis, but also hints that *CD63* can be a therapeutic target for kidney cancer with minimal systemic toxicity. Anti-*CD63* antibodies were reported to suppress allergy (*34*) or inhibit metastasis (*35*) in vivo. It will be worth exploring whether anti-*CD63* antibodies are able to improve the potency of targeted therapy or immunotherapy and inhibit metastasis in patients with kidney cancer. Nevertheless, this example suggests that the core genes selected by SCOPE may help exert bona fide biological functions in the mechanisms of cancer.

Among all genes connected with *CD63*, *SAT2* is the one with the most significantly differential correlations in tumor and normal tissues. *SAT2* mRNA level shows a negative correlation with CD63 in normal tissue while exhibiting an almost opposite correlation in tumors (Fig. 7E, 1 and 2). Many other genes in the pathway of arginine and proline metabolism, such as *AGMAT*, *DAO*, *ALDH4A1*, *PRODH2*, *GATM*, and *HOGA1*, also show similar patterns of switched correlations with *CD63* (Fig. 7E, 1 and 2). The altered correlations in tumors uncovered by SCOPE hint that these genes may play critical roles in kidney tumorigenesis. In agreement with this hypothesis, agmatinase, encoded by *AGMAT*, is diminished in kidney cancer samples, whereas *AGMAT* mRNA is most abundant in human liver and kidney (*36*). Moreover, *SAT2*, *DAO*, *ALDH4A1*, *PRODH2*, *GATM*, and *HOGA1* are ubiquitously expressed in the kidney based on the Human Protein Atlas (*37*, *38*). However, other genes belonging to the pathway of arginine and proline metabolism were not identified or were only shown negligible correlation differences by SCOPE, such as *SAT1*, *NOS1*, *CKM*, *CKB*, and *ARG2*, and do not show obvious overexpression in the kidney (*37*–*39*). The distinct tissue specificity of two groups of genes in the same pathway of arginine and proline metabolism validates that SCOPE was able to identify altered coexpression patterns in specific cancer types. In contrast, neither DE nor DiffCoEx uncovered significant enrichment of these pathways in KIRC, further strengthening the ability of SCOPE in uncovering such biologically relevant pathways.

## DISCUSSION

Current methods of driver gene identification use multiomics data, particularly mutation data in collaboration with known biological pathways. Transcriptomic data are seldom used for the identification of driver genes. This is in part due to the inability to determine causality using methods such as DE, DiffCoEx, and coexpression networks. Gene expression data alone, while conveniently available, are infrequently used for this purpose and rather directed toward biomarker discovery. Our proposed method of stabilizing the LASSO such that it identifies consistent predictors followed by coexpression and pathway analysis enables researchers to identify the core genes and pathways by taking advantage of the synergy between two disconnected fields: linear feature selection and nonlinear coexpression network analysis. This provides a method for experimentalists to narrow down candidate genes using more cost-effective expression data. Furthermore, only a handful of such core genes are selected, thus providing experimentalists an ideal scenario of being able to study these few genes extensively.

SCOPE uses both coexpression and DiffCoEx in building the CGNs that represent the units for enrichment. While it is usual for coexpression to be typically studied, DiffCoEx is less used and the combination even less so. The intuition here is that while genes coexpressed in both tumor and normal tissues are clearly interacting with the core genes identified, differentially coexpressed genes are even more so due to the differences in their behavior between the two phenotypes. Thus, combining both types of interactions leads to a better constructed network, identifying more interesting groups of genes to be studied.

Discovering enriched pathways connected with core genes may provide a rationale for targeted therapy against certain cancer types. A series of genes in the ferroptosis pathway, including *ACSL4*, *MAP1LC3B*, *ATG5*, *PRNP*, *NCOA4*, *PCBP1*, *LPCAT3*, *VDAC3*, *FTH1*, *SLC39A14*, *SLC40A1*, and *SLC11A2*, showed significantly changed patterns of correlation with *PSMG4* in the PRAD dataset (table S19). Ferroptosis is a programmed cell death driven by iron-dependent phospholipid peroxidation and reactive oxygen species generation (*40*). Since excessive iron contributes to ferroptosis, *PCBP1* and *FTH1*, which regulate iron metabolism and storage, are considered negative regulators of ferroptosis. *ATG5*, *MAP1LC3B*, and *NCOA4* initiate autophagy and consequently promote iron release from degraded iron-bound proteins. *SLC40A1*, *SLC39A14*, and *PRNP* export iron from cells and reduce ferroptosis, whereas *SLC11A2* regulates iron release to the cytoplasm and may enhance ferroptosis. *ACSL4*, *LPCAT3*, and *VDAC3* regulate the mechanism of phospholipid and *NADH* oxidation and play roles as positive regulators of ferroptosis (*41*). In particular, *AIFM2*, a critical ferroptosis suppressor identified in 2019 (*42*, *43*), shows reduced expression in prostate cancer (PRAD) [logFC (fold change) = −0.9008]. All these data indicate that ferroptosis inducers might be potent in patients with PRAD. Consistently, recent work has reported the induction of ferroptosis as a new therapeutic strategy for advanced prostate cancer (*44*). Neither DiffCoEx nor DE highlighted the ferroptosis pathway as significant in PRAD, while SCOPE was able to highlight this pathway uniquely and significantly in PRAD.

There are also many other pan-cancer analyses. A weighted gene co-expression network analysis (WGCNA) (*45*)–based approach (*46*) identified multiple hallmarks of cancer stratifying different tumors contrary to SCOPE, where the pathways and hallmarks that are shared by different cancers are identified. Another study conducting survival analysis based on the TCGA database (*47*) identifies unique prognostic tumor-specific genes that are also cancer hallmark genes and remarks on their tumor specificity. However, the shared pathways identified by SCOPE may highlight that cancer hallmarks may be induced at a pathway level even if the same hallmark genes are not clearly expressed in each type of tumor. A mutation-based approach to pan-cancer network analysis (*48*) identifies 16 significant subnetworks that span across multiple pathways with previously identified roles in cancer, further contributing to the hypothesis of shared pathways explored by SCOPE.

An inherent limitation of transcriptomic data is that most biological functions are performed by proteins, not mRNAs. One example is the p53 signaling pathway in BRCA, which is significantly enriched by the gene pairs of *MT-CO2* with *CDK4*, *AIFM2*, or *CHEK2* (table S20). Furthermore, another core gene, *CD300LG*, shows switched correlations with *TP53* and *CASP9*, although the respective CGN is not enriched for the p53 signaling pathway. Although *TP53* (encoding p53) showed altered correlations with *CD300LG* and *MT-CO2*, the putative transcriptional targets of p53, such as *CDKN1A* and *MDM2* (*49*), did not show significant changes of correlation. It implies that the p53 transcriptional activity was not significantly changed in the presence of significantly changed mRNA level of *TP53*. This implication was further supported by two facts: (i) The regulation of p53 activity is dominant at the posttranslational level (*49*), not at the mRNA level; (ii) 35% of patients in the TCGA-BRCA database harbor *TP53* mutations, and most *TP53* mutations abolish the transcriptional activity of p53. We looked for top transcription factor binding sites in the promoters of these genes (*CASP9*, *CDK4*, *AIFM2*, and *CHEK2*) provided by QIAGEN through GeneCards (*50*) and found CCAAT/enhancer binding proteins (C/EBPs) bound to these promoters. Since the phosphatidylinositol 3-kinase (PI3K)–AKT–mTOR signaling pathway is highly mutated in the BRCA database [fig. S17; obtained from cBioPortal in the TCGA-BRCA database (*51*, *52*)] and is able to regulate the transcriptional activity of C/EBPs (*53*), a reasonable explanation is that a hyperactivated PI3K-AKT-mTOR axis induces the mRNA expression of these targets via C/EBPs as the transcriptional factor (but not *TP53*) in patients with BRCA. Nevertheless, with more data of cancer at the protein level, such as The Pathology Atlas (*38*) and The Cancer Proteome Atlas Portal (*54*, *55*), SCOPE may be substantially empowered to provide more valuable insights into the aberrant connections in tumor cells.

To recap, we have presented SCOPE, a method stabilizing gene selection and coexpression network analysis, which is able to identify core genes and pathways underlying cancers. Its effectiveness has been demonstrated by various analyses from three angles (i.e., selection of few, stable, and predictive genes; pan-cancer shared pathways; and the role of core genes in connectivity analysis). Moreover, in-depth annotations have revealed the pivotal role of *CD63* on tumorigenesis in kidney cancer and the potential therapeutic application of anti-*CD63* antibody on patients with kidney cancer. As a proof of concept, we have only contrasted cancer and normal tissues in this work. However, the statistical framework is applicable to any case/control settings. In the future, we will adapt SCOPE to analyze clinically important qualities such as whether a patient will respond to medical treatments such as immunotherapy, paving the way to the application of precision medicine in more applications.

## MATERIALS AND METHODS

### SCOPE-Stabilized LASSO selection

While LASSO has proven versatile in many applications, statistically, it has become apparent that in the presence of multiple correlated features, it may be inconsistent in its selection of features, even in multiple random samplings of the same data (*56*). This has led to a number of new methods being proposed that are all modifications of the original LASSO such as adaptive LASSO (*56*), random LASSO (*57*), and bolasso (*19*). A seminal work by Meinshausen and Bühlmann (*17*) discusses stability paths, which obtain the selection probabilities of each feature by subsampling along all possible values of the tuning parameter with randomized LASSO, which introduces a random penalty λ for each feature. While such methods are statistically proven and can lead to sound results, they appear to be seldom used in the field of genomics.

In SCOPE, a simpler solution to the inconsistency of variable selection in LASSO is proposed and applied in the form of a bootstrapped LASSO (Fig. 1, A and B). While simple in design, it produces consistent results that are highly predictive. In the case of this paper, where the phenotype is binary (tumor or normal sample), a logistic LASSO regression model is trained multiple times by subsampling from the same dataset. Genes that were selected in most of the models (over a threshold proportion θ_thr_) are used to build a final logistic regression model for which the final accuracy will be assessed. This stable subset of genes is proposed to be the “core” genes of the disease. These core genes can then be used in other predictive models or for further downstream analysis; SCOPE uses a coexpression-based pathway analysis using these selected core genes.

The SCOPE-Stabilized LASSO used in this analysis features the consensus of 1000 training-test splits of a 70%-30% split ratio (each with a consistent case/control ratio of the full dataset). Each LASSO model trained was tuned for the optimal value of λ using 10-fold cross-validation. The thresholds used for the different datasets of the real analysis are detailed in Results.

### Coexpression and pathway analysis

It is assumed that core genes interact with multiple other genes that may be involved in pathways responsible for disease mechanisms. To identify these genes, we conducted both coexpression and DiffCoEx analysis (Fig. 1D). To claim a gene as being significantly coexpressed with a core gene, we required a null distribution for the correlations (coexpression) between pairs of random genes. To this end, we drew random pairs of genes and calculated the Pearson correlation coefficients of these pairs. Using this distribution, we obtain the (*r*_thr_=) 97.5th percentiles for both positive and negative correlations. This allowed us to identify genes that are significantly coexpressed with core genes. Each set of genes thus identified (secondary coexpressed genes, along with their corresponding core gene), termed CGNs, was then tested for pathway enrichment.

To reflect the fact that some critical genes are not so highly coexpressed but are significantly differentially coexpressed when contrasting cancer and normal tissues, we also obtained the genes that are significantly differently coexpressed with the core gene between tumor and normal tissues (Fig. 1D). As in the case of the coexpression analysis, a null distribution of the DiffCoEx values (|corr_case_ − corr_control_|) was obtained, and the ($r_{\mathrm{thr}}^{D}=$) 97.5th percentile was used to select significantly differentially coexpressed secondary genes. These secondary genes from DiffCoEx analysis were also added to the CGNs for pathway analysis below.

Pathway enrichment is typically used to assess whether a particular set of genes overlap with known biological pathways significantly higher than by chance. There are many databases containing such pathways, and SCOPE uses the KEGG (*58*, *59*) database because of its comprehensiveness and popularity. Overrepresentation analysis (*60*) is used to identify the statistical significance, and the R package WebGestaltR (*61*, *62*) was used for testing pathway enrichment against the KEGG database. This analysis results in several pathways enriched (at FDR ≤ 0.05) for each CGN (comprising of genes both coexpressed and differentially coexpressed) underlying the focal core gene. SCOPE then discovers pathways that are commonly influenced by CGNs (seeded by different core genes).

For a single disease such as a cancer, an index for the level of sharing of a pathway (across multiple CGNs within a cancer), π_cancer_, is defined as the number of coexpressed genes (including the core gene) found to be enriched in this pathway divided by the total number of genes in the pathway. When multiple diseases are jointly analyzed (e.g., the six cancers used here as a demonstrating example), SCOPE will further discover pathways common to all diseases (table S17). The summation of this single cancer-specific index (π_cancer_) over all the cancers is noted as the POS. Intuitively, a higher POS indicates a higher overlap of the pathway across different cancers.

### Methods compared to SCOPE

#### Least absolute shrinkage and selection operator

The primary benchmark and point of comparison is a traditional L_1_ regularized logistic regression model, which uses the addition of the absolute value of the coefficients to promote sparsity in the loss function. Given *n* number of samples and *p* number of features/variables, the regularized loss function of a logistic regression model takes the form

$$\min_{\beta }\sum_{i=1}^{n}[y_{i}x_{i}^{T}\beta -\log(1+e^{x_{i}^{T}\beta })]+\lambda \sum_{j=1}^{p}|\beta_{j}|$$ (1)

where λ is the tuning parameter controlling the trade-off between sparsity and accuracy. Ten-fold cross-validation is typically used to tune for this parameter, and the R package glmnet (*63*, *64*) enhanced by glmnetUtils (*65*) was used to fit LASSO models here (Supplementary Materials). Genes selected by a traditional LASSO and genes selected by the SCOPE-Stabilized LASSO step are compared in Results.

#### Adaptive Elastic-Net

The Adaptive Elastic-Net (*23*) is essentially a merging of the popular elastic-net (*66*) (which combines L_1_ and L_2_ regularization) and the adaptive LASSO (*56*), which assigns data-dependent weights to the coefficients in the L_1_ penalty. The data-dependent weights are calculated using a standard elastic-net model, and these estimates are denoted by $\hat{\beta }(\mathrm{enet})$. The Adaptive Elastic-Net estimates are then given by

$$\hat{\hat{\text{β}}}=\left(1+\frac{\lambda_{2}}{n}\right)\left\{\underset{\beta }{\text{arg min}}{||y-X\text{β}||}_{2}^{2}+\lambda_{2}{||\text{β}||}_{2}^{2}+\lambda_{1}^{*}\sum_{j=1}^{p}{\hat{w}}_{j}|\beta_{j}|\right\}$$

where ${\hat{w}}_{j}={\left[|{\hat{\beta }}_{j}(\mathrm{enet})|\right]}^{-\gamma }$ and γ is a positive constant. The R package gcdnet (*67*) is used to tune and fit the Adaptive Elastic-Net models used here, while the elastic-net weights were obtained using the glmnet package.

#### Randomized LASSO

The randomized LASSO modifies the penalty λ of a typical LASSO model to a randomly chosen value in the range $\left[\lambda ,\frac{\lambda }{\alpha }\right]$ where α ∈ (0,1] (default = 0.8) is defined as the weakness. Assuming *W_j_* to be independent, identically distributed (IID) random variables in [α,1], the randomized LASSO estimator is

$$\hat{\hat{\text{β}}}=\underset{\text{β}}{\text{arg min}}\left({||y-X\text{β}||}_{2}^{2}+\lambda \sum_{j=1}^{p}\frac{|\beta_{j}|}{W_{j}}\right)$$

Randomized LASSO, as described in (*17*), also follows the additional step of stability selection by selecting only variables that are above a certain threshold (π_thr_, default = 0.8) across random subsamples. The implementation of randomized LASSO in the monaLisa (*68*) R package was used for comparison purposes here.

#### DiffCoEx analysis

Standard network-based methods of analysis of expression data typically use the interconnectedness of genes (in the form of coexpression) to identify important networks (or “modules”) of genes. However, in a case-control setting, the use of DiffCoEx can prove more informative because of the contrastive nature of the analysis. DiffCoEx (*69*), one of the most popular methods extending the popular WGCNA coexpression network analysis tool, was chosen for comparison. DiffCoEx identified differentially coexpressed modules (Supplementary Materials) that were used in pathway enrichment similar to how CGNs were used for pathway enrichment and for studying pathway overlaps.

#### Differential expression

An edgeR-limma–based pipeline (*70*) was used to normalize the data to log_2_–counts per million values, and a linear model incorporating weights from voom to correct for the mean-variance relationship was used to statistically detect the DE of genes in each of the cancers. The pipeline was run using default values for all parameters as described in the workflow.

### Data generation and model fitting for simulations

A number of different factors such as signal-to-noise ratio, linear/nonlinear effects on phenotype, and different coexpression structures were considered in generating the data required for the simulations. The GTEx (*22*) was used as the source of expressions. A random sample of 15 pathways from the KEGG pathway database was considered as candidates of gold-standard core pathways. Each simulation begins by randomly sampling *p* (*p* = 3,5,10) number of core pathways from the available pathways. Then, the Pearson correlation of all genes in the 15 pathways is calculated pairwise for each of the genes in the core pathways. The absolute sums of these values are then calculated, providing an empirical estimate of the interaction of each gene with the core pathways. Then, *g*_c_ (*g*_c_ = 5,10,15) number of core genes are selected on the basis of (i) the highest interacting genes and (ii) the lowest interacting genes with the core pathways. A further *g*_e_ (*g*_e_ = 0,3,5) extra causal genes are randomly selected from all the remaining genes of the 15 pathways, and the combined set of genes, {*g*_c_, *g*_e_}, was used to generate the phenotype.

Phenotypes are generated using both linear and nonlinear models:

1) Linear: $Y_{\mathrm{initial}}^{\mathrm{linear}}=\sum_{j=1}^{g_{c}+g_{e}}\beta_{j}X_{j}$, where β~Unif( − 10,10)

2) Nonlinear: $Y_{\mathrm{initial}}^{\mathrm{nonlinear}}=\sum_{j=1}^{g_{c}+g_{e}}\sum_{i=1}^{j}\beta_{j,i}X_{j}⊙X_{i}$, where **β**~Unif( − 10,10) and ⊙ represents element-wise multiplication

To ensure that a consistent signal-to-noise ratio (*s*_nr_ = 0.7,0.8,0.9) is achieved and a binary phenotype is produced, both *Y*_initial_ values undergo the following transformations to obtain the respective phenotypes. Let $\sigma_{g}^{2}=\mathrm{var}(Y_{\mathrm{initial}})$. Then, $\sigma_{\mathrm{error}}^{2}=\sigma_{g}^{2}\left[\frac{1}{s_{\mathrm{nr}}^{2}}-1\right]$ and ***Y***_noisy_ = ***Y***_initial_ + ϵ where $\text{ϵ}∼N(0,\sigma_{\mathrm{error}}^{2})$. Then, let $p=\frac{1}{1+\exp(-Y_{\mathrm{noisy}})}$. Last, *Y_i_*~*Bin*(1, *p_i_*). Ten replicates of each unique combination of parameters were obtained, resulting in a total of 3240 simulations.

The above procedure generates data ready for analysis. The analytic procedure is generally the same as what we did for real data analysis. The only alteration is on the interaction term in the nonlinear case. In practice, one has to rely on regularized regression to select causal genes out of many candidates. Following this procedure, when analyzing the data, around 700 terms (genes) from the randomly selected pathways were included in the regularized regression to test the ability of feature selections. However, in the nonlinear cases, if we put all potential combinations of all noisy genes to the model, the power will be close to zero. As such, we assume that the candidates under interactions are known and only put the interaction terms between these genes into the regression. This might still be close to the practice as users should have a rough idea of which genes are under interactions; without which, such feature selection methods would not work well. Nevertheless, we make the interacting genes available for all three competitive methods to ensure the fairness of the comparison.

### Real data analysis

#### Data source and processing

The TCGA program initiated by the National Cancer Institute and the National Human Genome Research Institute in 2006 (*20*) offers a wealth of omics data on 32 different cancers and their subtypes at 68 primary sites. This includes RNA sequencing data that provide a snapshot of the transcriptomic landscape of the tumor site and of solid normal tissue close to the tumor site.

Six cancers [breast invasive carcinoma (BRCA), kidney renal clear cell carcinoma (KIRC), lung adenocarcinoma (LUAD), colon adenocarcinoma (COAD), prostate adenocarcinoma (PRAD), and thyroid carcinoma (THCA)] were chosen primarily because of the large samples of expression data available and the inclusion of normal tissue from the same patients. A breakdown of the sample sizes and disease status is given in table S21. The raw count data were downloaded from the TCGA data portal and then converted to transcripts per million values using gene lengths obtained through the biomaRt (*71*, *72*) package. Phenotype (tumor or normal) was determined on the basis of the sample type column provided in the database, and “primary tissue” was considered as cases and normal tissue as controls. Any other sample types such as “metastasis” were discarded. Models were then fitted on these processed data. Two additional datasets (lung cancer associated), E-MTAB-6043 (*24*) and MTAB-6699 (*25*), were downloaded from ArrayExpress and used to externally validate the predictive accuracy of core genes selected by SCOPE and alternative methods.

## Notes

View/request a protocol for this paper from *Bio-protocol*.
