# Supplementary material for: Stabilized COre gene and Pathway Election uncovers pan-cancer shared pathways and a cancer-specific driver
Source: Sci Adv. 2022 Dec 21;8(51):eabo2846. doi: 10.1126/sciadv.abo2846 (PMC9770999; doi:10.1126/sciadv.abo2846)
Supplement: Supplementary file 1 — Supplementary Text Figs. S1 to S17 Tables S1 to S16, S19 and S21 [file sciadv.abo2846_sm.pdf]

Science Advances
AAAS

Supplementary Materials for

Stabilized COre gene and Pathway Election uncovers pan-cancer shared pathways and a cancer-specific driver

Pathum Kossinna *et al.*

Corresponding author: Qingrun Zhang, qingrun.zhang@ucalgary.ca

Sci. Adv. **8**, eabo2846 (2022)
DOI: 10.1126/sciadv.abo2846

The PDF file includes:

Supplementary Text
Figs. S1 to S17
Tables S1 to S16, S19 and S21
Legends for tables S17, S18 and S20

Other Supplementary Material for this manuscript includes the following:

Tables S17, S18 and S20

Supplementary Text

SCOPE Algorithm (Extended)

Input: Expression Data $\mathbf{X}_{n\times p}$

Phenotype Data $\mathbf{Y}_{n\times 1}$

Number of splits m

Proportion of selection threshold θ_{thr}

Stabilized LASSO

for $i = 1$ to m

Generate a random train-test split

Calculate LASSO estimates β_{ji} for split i

end for

Compute $\theta_{\beta_j} = \frac{\left|\sum_{i=1}^{m} I(\beta_{ji} \neq 0)\right|}{m}$, where I(.) is an indicator function

Select core genes $CGs = \left\{gene_j \middle| \theta_{\beta_j} \geq \theta_{thr}\right\}$

Co-expression Analysis

for $i = 1$ to 1000

Select 1000 random genes

Calculate correlation values

$\boldsymbol{corrs^{+}_{thr}}, \boldsymbol{corrs^{-}_{thr}}.\mathrm{append}($97.5th percentile of positive and negative correlations)

end for

$corr^{+}_{thr}, corr^{-}_{thr} = \mathrm{medians}(corrs^{+}_{thr}, corrs^{-}_{thr})$

for each $\boldsymbol{CG_i}$

Calculate $corr_{i,j}$ ∀ other genes j

if $corr_{i,j} > corr^{+}_{thr}$ OR $corr_{i,j} < corr^{-}_{thr}$ **then**

$gene_j$ is co-expressed with core gene i

$\mathrm{CGN}_i.\mathrm{append}(gene_j)$

end for

Repeat for Differential Co-expression

Pathway Enrichment

Input: $\mathrm{CGN}_i\ \forall i$

for each $\mathbf{CGN}_{\boldsymbol{i}}$

$P_i = \{p | p \text{ enriched in } \mathrm{CGN}_i\}$

end for

$\forall\, p_j, PS_j = \sum_{i=1}^{|CG|} I(p_j \in P_i)$

Output:

Pathways enriched for each CGN_i, $\boldsymbol{P_i}$

Number of CGNs enriching each pathway p_j, $\boldsymbol{PS_j}$

LASSO (Least Absolute Shrinkage and Selection Operator)

Among multiple methods proposed for variable selection and model building, the LASSO (*18*) and ridge (*15*) regularized regression methods have gained popularity in recent years for high-dimensionality problems. Regularization typically refers to the addition of an additional term in the loss function that is meant to prevent overfitting. LASSO (a form of L_1 regularization) adds the sum of absolute values of the regression coefficients to the loss function and in turn allows some of these coefficients to be reduced to 0 – thus inducing sparsity. It has been shown that L1 regularization promotes sparsity more than higher order regularizations (such as L2, or ridge regression) among convex forms of regularization models (*18*).

In particular, a typical regularized loss function is:

$$\min_{\beta} \sum_{i=1}^{n} (y_i - x_{\mathrm{i}}^T \beta)^2 + \lambda \sum_{j=1}^{p} |\beta_j| \, ,$$

where λ is a non-negative tuning parameter that controls the trade-off between sparsity and accuracy, n is the number of samples and p is the number of features/variables.

In this paper, a logistic regression model is used and therefore, the loss function becomes,

$$\min_{\beta} \sum_{i=1}^{n} \left[y_i x_i^T \beta - \log\left(1 + e^{x_i^T \beta}\right) \right] + \lambda \sum_{j=1}^{p} |\beta_j|.$$

The tuning parameter λ is typically chosen via cross-validation and in this paper, a 10-fold cross validation is used to select the optimal λ.

Differential Co-expression Analysis (DiffCoEx)

To compare our method with a standard method based on network analysis, we use differential co-expression networks to identify groups (or "modules") of differentially co-expressed genes and conduct pathway enrichment on these modules. While there are multiple methods of differential co-expression analysis, the most widely used method remains DiffCoEx (*70*), an extension of the popular WGCNA (*46*) to differential co-expression. DiffCoEx begins with the construction of two adjacency matrices: C_{case}: $c_{ij}^{case} = cor(gene_i, gene_j)$ for cancerous samples and $C_{control}$ similarly for healthy samples.

While different correlation measures can be used in this step, the authors of DiffCoEx used the Spearman rank correlation. A matrix of adjacency difference is then calculated,

$$D: d_{ij} = \left(\sqrt{\frac{1}{2} \left| sign\left(c_{ij}^{case}\right) * \left(c_{ij}^{case}\right)^2 - sign\left(c_{ij}^{control}\right) * \left(c_{ij}^{control}\right)^2 \right|} \right)^{\beta}$$

where $\beta \geq 0$ is an integer tuning parameter which can be selected in multiple ways. In this paper we choose $\beta \in [5,6,7,8,9,10]$ such that each cancer has the minimum number of modules with the largest module containing the smallest number of genes. Next, a Topological Overlap dissimilarity Matrix (TOM) is calculated where lower values of t_{ij} indicate that a pair of genes

$gene_i$ and $gene_j$ have significant correlation changes (between case and control) with the same group of genes.

$$T: t_{ij} = 1 - \left(\frac{\sum_k d_{ik} d_{kj} + d_{ij}}{\min\left(\sum_k d_{ik}, \sum_k d_{jk}\right) + 1 - d_{ij}} \right)$$

Finally, the dissimilarity matrix T is used for clustering and “modules” of differentially co-expressed genes are identified. These modules which contain sets of genes are then tested for pathway enrichment and the same measures of overlap as in the SCOPE method are calculated.

Supplementary Figure S1 – Impact of tuning parameters on Pathway Overlap Score

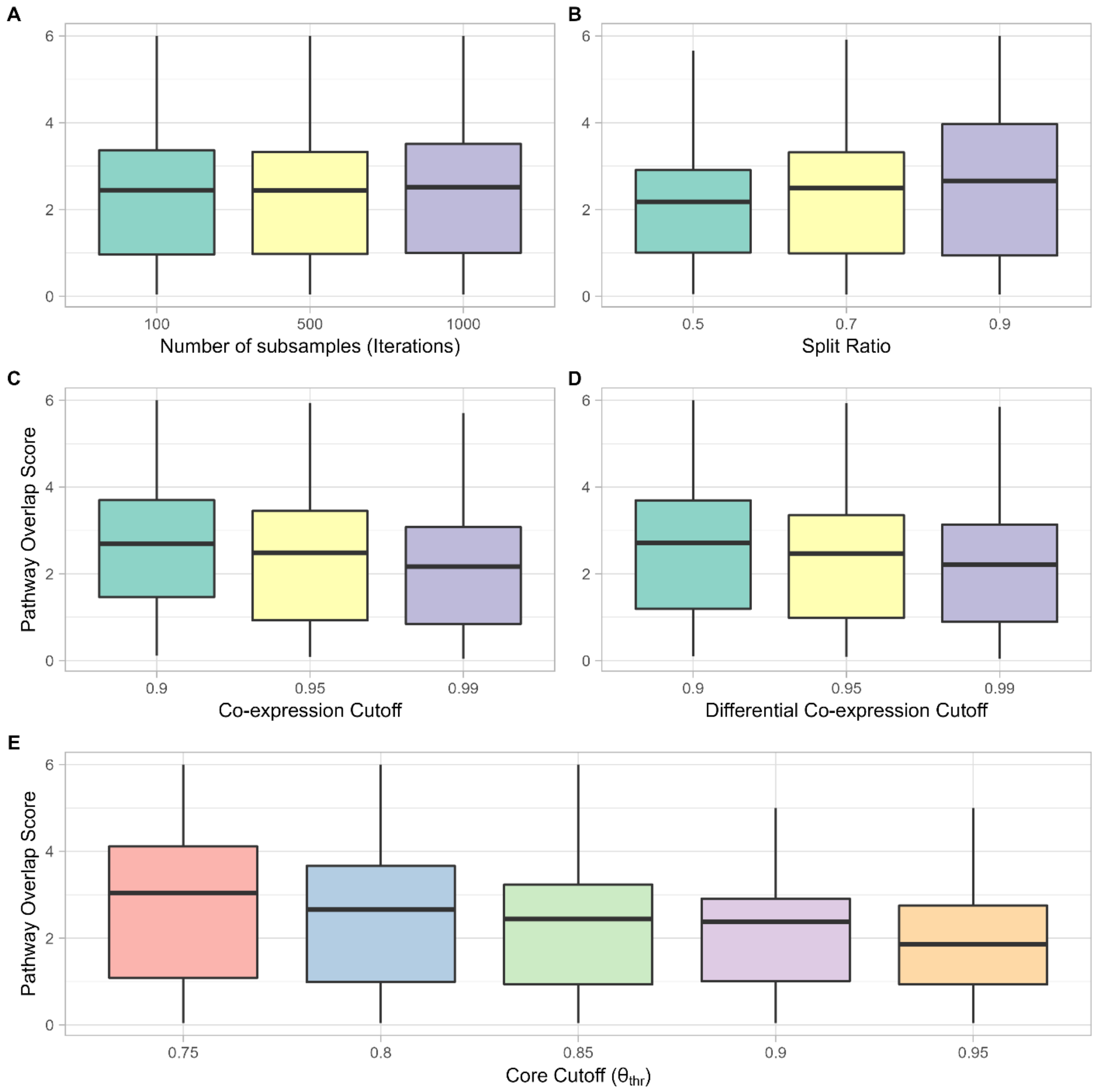

Range of Pathway Overlap Scores (POS) across the six cancers (BRCA, COAD, KIRC, LUAD, PRAD and THCA) of the TCGA database when running SCOPE using multiple sets of values for the parameters indicates similar maximum POS scores for different values of the **A)** number of iterations/subsamples (n_{iter}), **B)** ratio of data used in each subsample (s_{prop}), **C)** co-expression cut-off (r_{thr}) and **D)** differential co-expression cut-off (r^D_{thr}). **D)** Indicates that POS Scores do change significantly when higher θ_{thr} values are specified (which also inversely relates to the number of core genes identified) leading to the conclusion that θ_{thr} may be tuned based on feasibility of further study of the core genes.

Supplementary Figure S2 – F1 Scores of Core Pathway identification of simulated gene expression data with 670 samples at FDR < 0.05

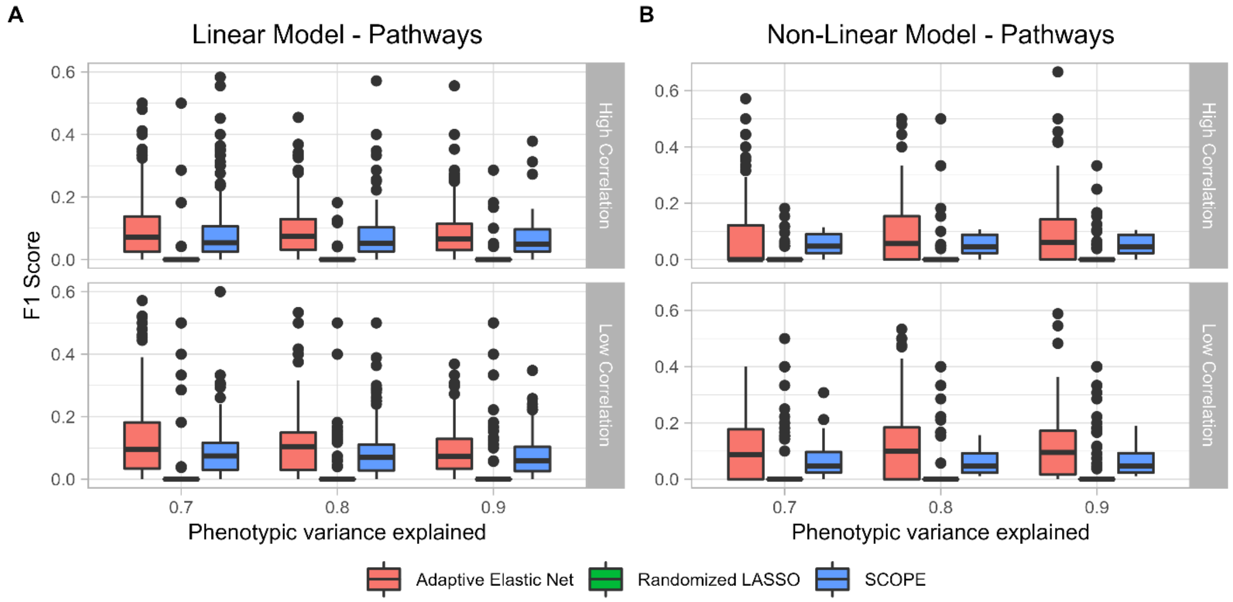

F1 Score (=TP/(TP+0.5*(FP+FN))) calculated for the accuracy of Adaptive Elastic-Net, Randomized LASSO and SCOPE models in identifying core pathways simulated in gene expression data with 670 samples at an FDR of <0.05 for both **A)** linearly generated phenotypes and **B)** non-linearly generated phenotypes with core genes of both high and low correlations individually. **Note:** In the non-linear model, we assumed that the genes participating interactions are known as a priori; otherwise, the powers of all three methods are close to zero. Please see detailed justifications in **Methods**.

Supplementary Figure S3 – F1 Scores of Core Gene identification of simulated gene expression data with 670 samples

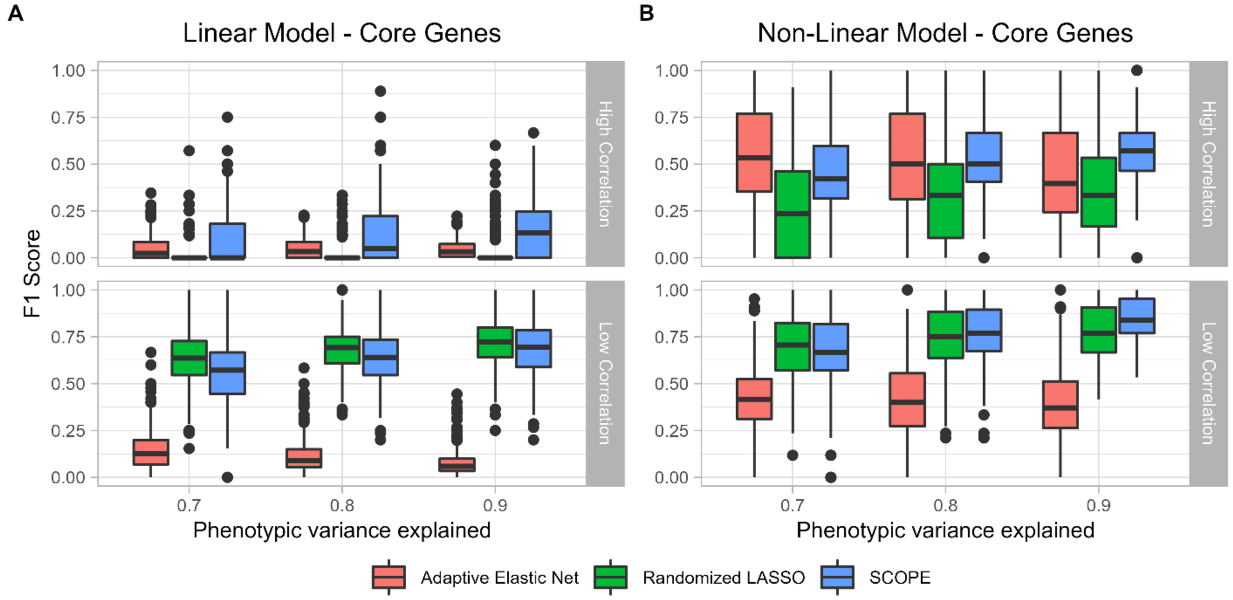

F1 Score (=TP/(TP+0.5*(FP+FN))) calculated for the accuracy of Adaptive Elastic-Net, Randomized LASSO and SCOPE models in identifying core genes simulated in gene expression data with 670 samples for both **A)** linearly generated phenotypes and **B)** non-linearly generated phenotypes with core genes of both high and low correlations individually. **Note:** In the non-linear model, we assumed that the genes participating interactions are known as a priori; otherwise, the powers of all three methods are close to zero. Please see detailed justifications in **Methods**.

Supplementary Figure S4 – Simulations comparing performance of SCOPE, Adaptive Elastic-Net and Randomized LASSO at a sample size of 500

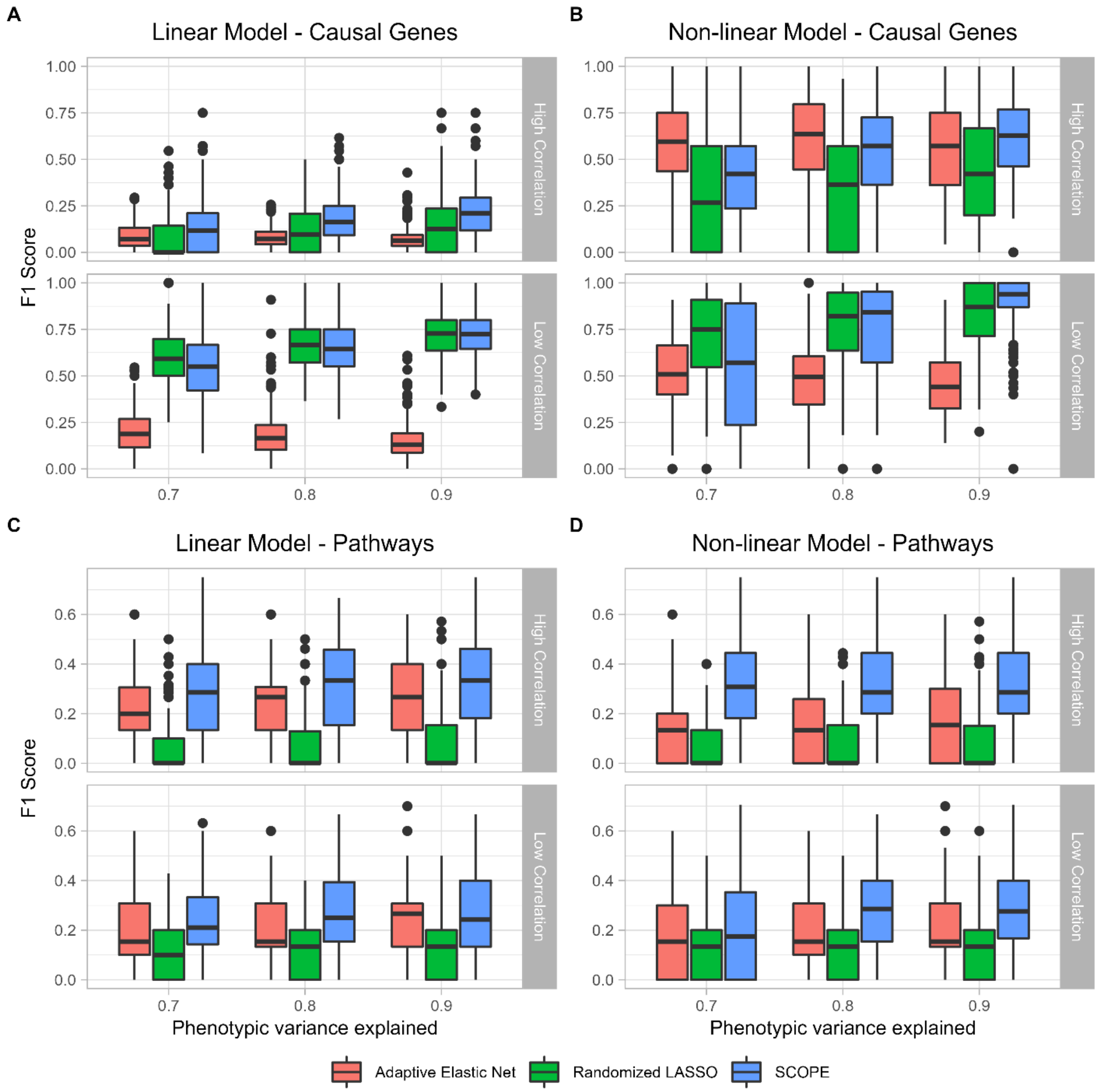

F1 Score (=TP/(TP+0.5*(FP+FN))) calculated for the accuracy of Adaptive Elastic-Net, Randomized LASSO and SCOPE models in identifying causal genes and pathways simulated in gene expression data with 500 samples. **A)** and **B)** indicate the ability of the three methods in identifying causal genes in linear and non-linear scenarios respectively under various correlation structures and signal-to-noise ratios. **C)** and **D)** indicate the ability of the three methods in identifying core pathways among the top 10 enriched pathways in linear and non-linear scenarios respectively under various correlation structures and signal-to-noise ratios. **Note:** In the non-linear model, we assumed that the genes participating interactions are known as a priori; otherwise, the powers of all three methods are close to zero. Please see detailed justifications in **Methods**.

Supplementary Figure S5 – F1 Scores of Core Pathway identification of simulated gene expression data with 500 samples at FDR < 0.05

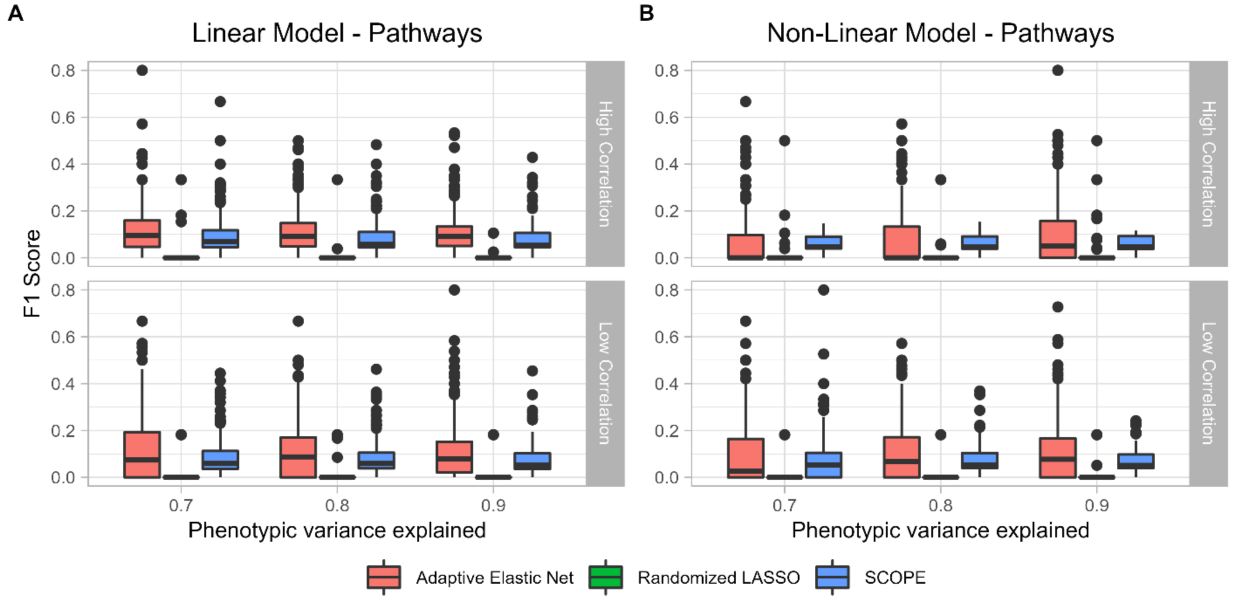

F1 Score (=TP/(TP+0.5*(FP+FN))) calculated for the accuracy of Adaptive Elastic-Net, Randomized LASSO and SCOPE models in identifying core pathways simulated in gene expression data with 500 samples at an FDR of <0.05 for both **A)** linearly generated phenotypes and **B)** non-linearly generated phenotypes with core genes of both high and low correlations individually. **Note:** In the non-linear model, we assumed that the genes participating interactions are known as a priori; otherwise, the powers of all three methods are close to zero. Please see detailed justifications in **Methods**.

Supplementary Figure S6 – F1 Scores of Core Gene identification of simulated gene expression data with 500 samples

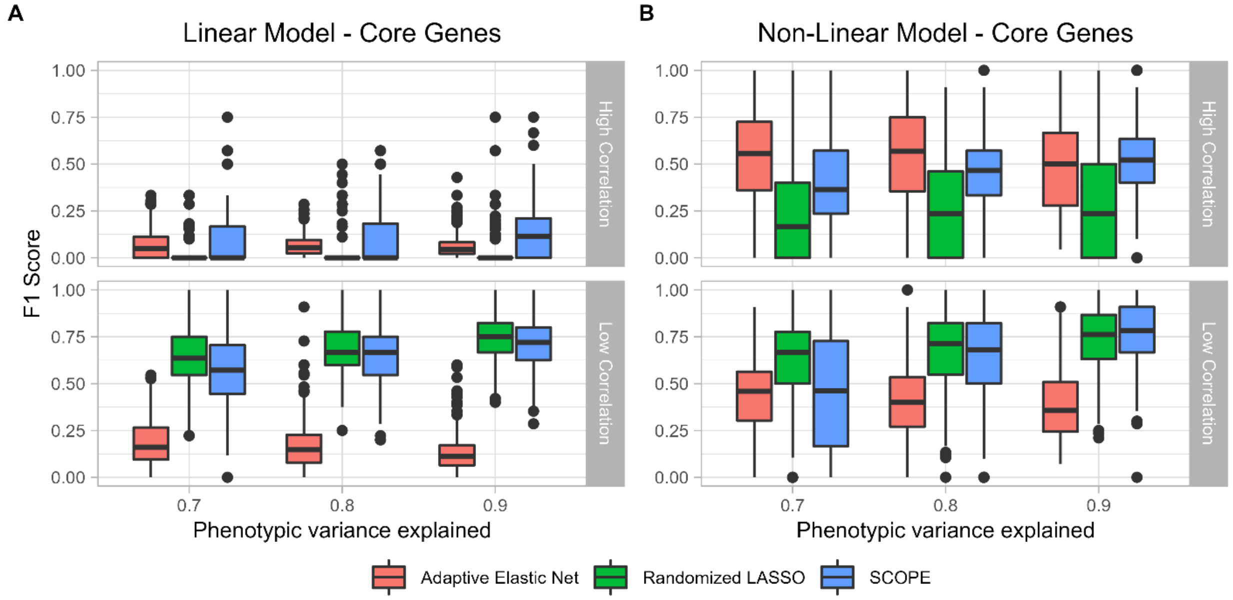

F1 Score (=TP/(TP+0.5*(FP+FN))) calculated for the accuracy of Adaptive Elastic-Net, Randomized LASSO and SCOPE models in identifying core genes simulated in gene expression data with 500 samples for both **A)** linearly generated phenotypes and **B)** non-linearly generated phenotypes with core genes of both high and low correlations individually. **Note:** In the non-linear model, we assumed that the genes participating interactions are known as a priori; otherwise, the powers of all three methods are close to zero. Please see detailed justifications in **Methods**.

Supplementary Figure S7 – Simulations comparing performance of SCOPE, Adaptive Elastic-Net and Randomized LASSO at a sample size of 250

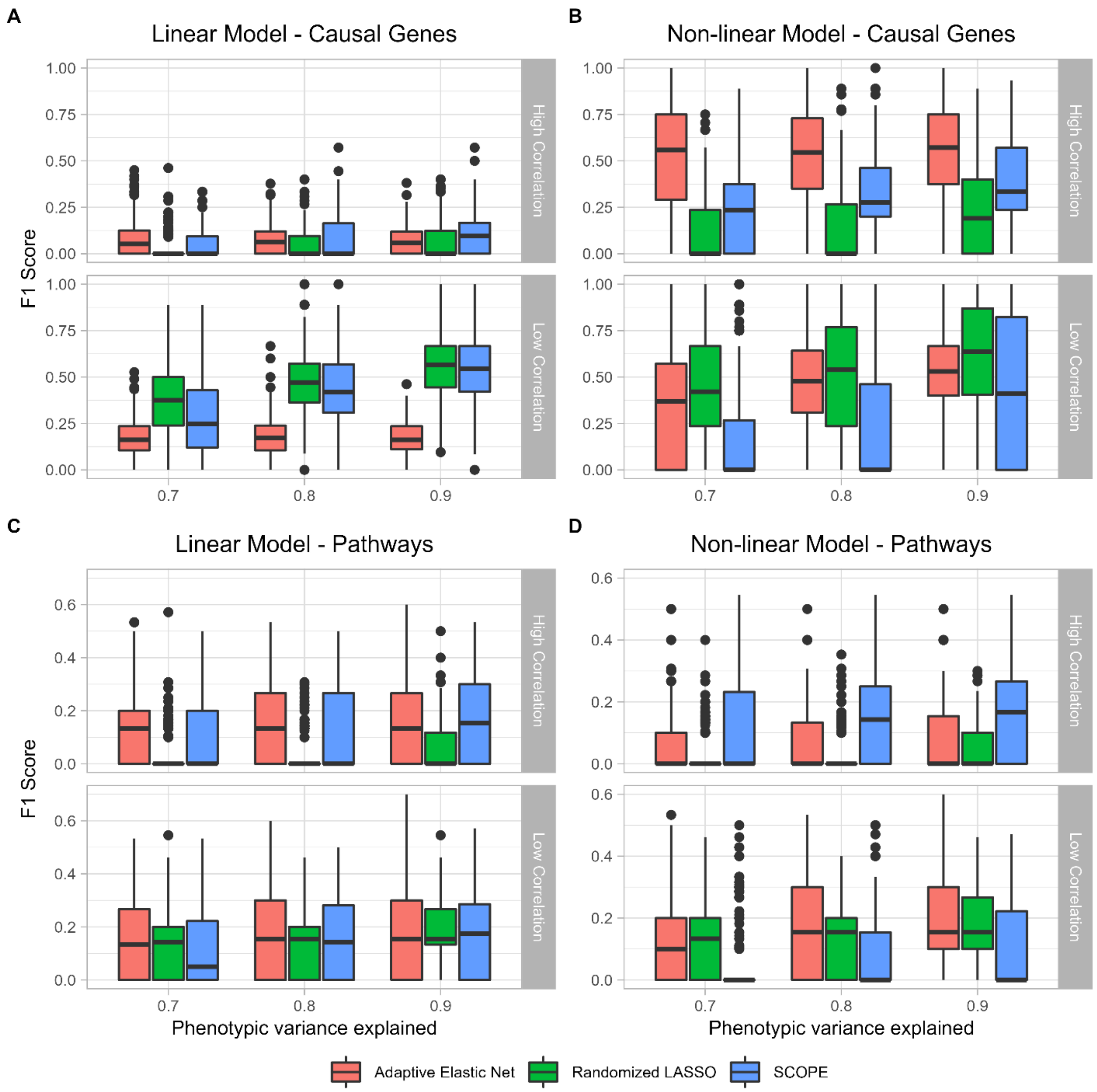

F1 Score (=TP/(TP+0.5*(FP+FN))) calculated for the accuracy of Adaptive Elastic-Net, Randomized LASSO and SCOPE models in identifying causal genes and pathways simulated in gene expression data with 670 samples. **A)** and **B)** indicate the ability of the three methods in identifying causal genes in linear and non-linear scenarios respectively under various correlation structures and signal-to-noise ratios. **C)** and **D)** indicate the ability of the three methods in identifying core pathways among the top 10 enriched pathways in linear and non-linear scenarios respectively under various correlation structures and signal-to-noise ratios. **Note:** In the non-linear model, we assumed that the genes participating interactions are known as a priori; otherwise, the powers of all three methods are close to zero. Please see detailed justifications in **Methods**.

Supplementary Figure S8 – F1 Scores of Core Pathway identification of simulated gene expression data with 250 samples at FDR < 0.05

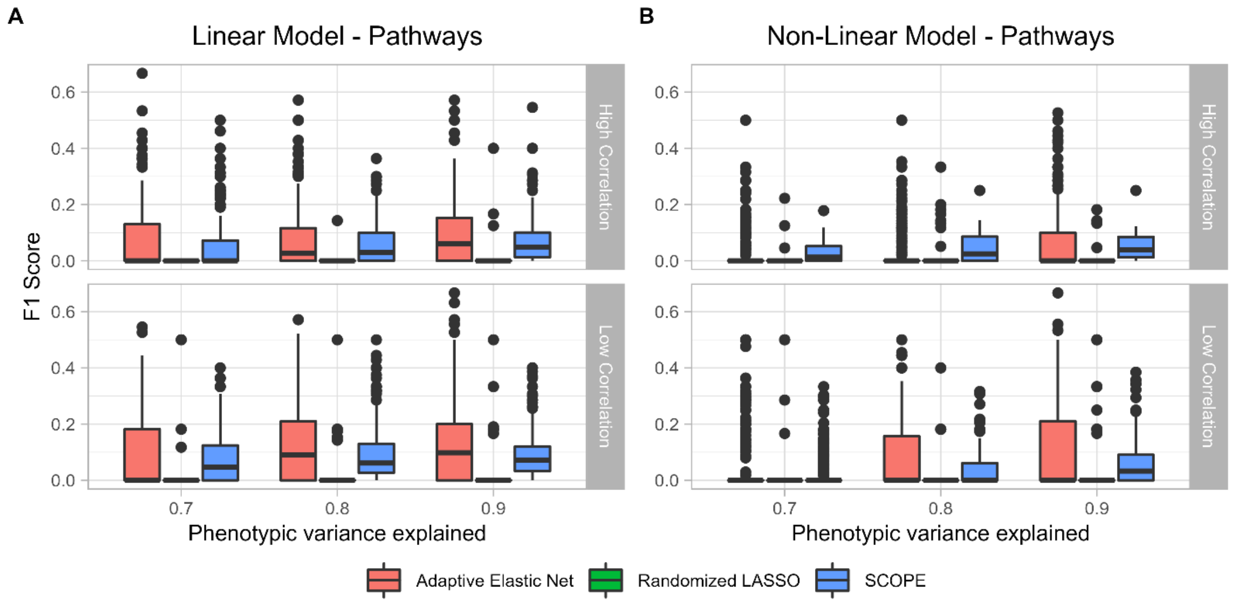

F1 Score (=TP/(TP+0.5*(FP+FN))) calculated for the accuracy of Adaptive Elastic-Net, Randomized LASSO and SCOPE models in identifying core pathways simulated in gene expression data with 250 samples at an FDR of <0.05 for both **A)** linearly generated phenotypes and **B)** non-linearly generated phenotypes with core genes of both high and low correlations individually. **Note:** In the non-linear model, we assumed that the genes participating interactions are known as a priori; otherwise, the powers of all three methods are close to zero. Please see detailed justifications in **Methods**.

Supplementary Figure S9 – F1 Scores of Core Gene identification of simulated gene expression data with 250 samples

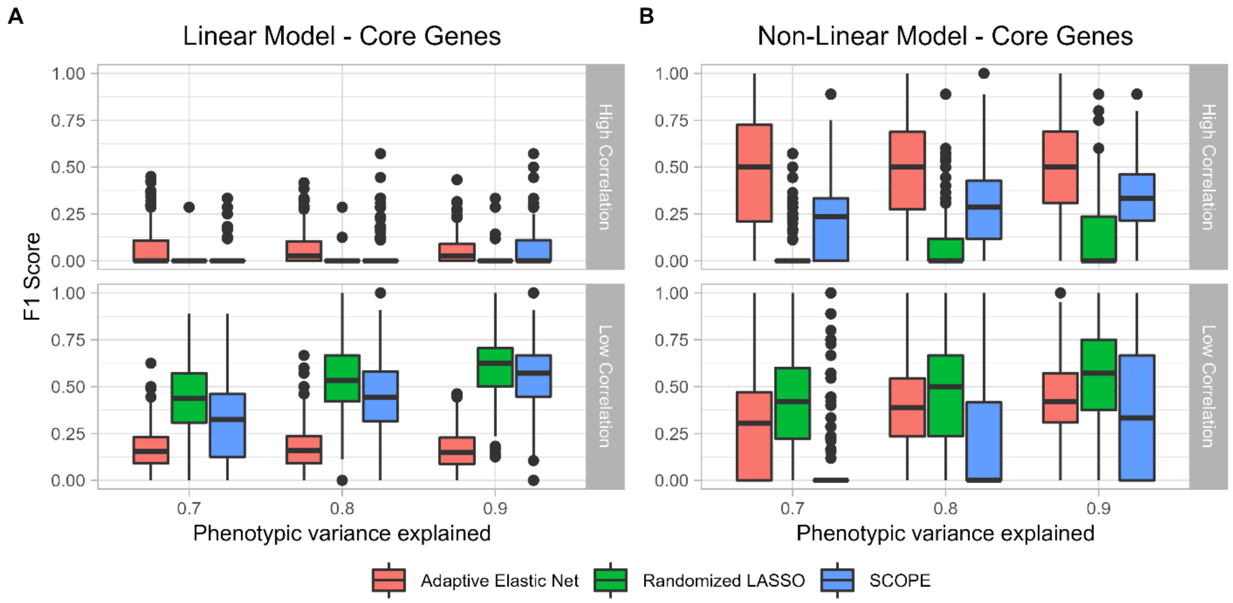

F1 Score (=TP/(TP+0.5*(FP+FN))) calculated for the accuracy of Adaptive Elastic-Net, Randomized LASSO and SCOPE models in identifying core genes simulated in gene expression data with 250 samples for both **A)** linearly generated phenotypes and **B)** non-linearly generated phenotypes with core genes of both high and low correlations individually. **Note:** In the non-linear model, we assumed that the genes participating interactions are known as a priori; otherwise, the powers of all three methods are close to zero. Please see detailed justifications in **Methods**.

Supplementary Figure S10 - DNA replication (hsa03030) and some core gene interactions highlighted by SCOPE.

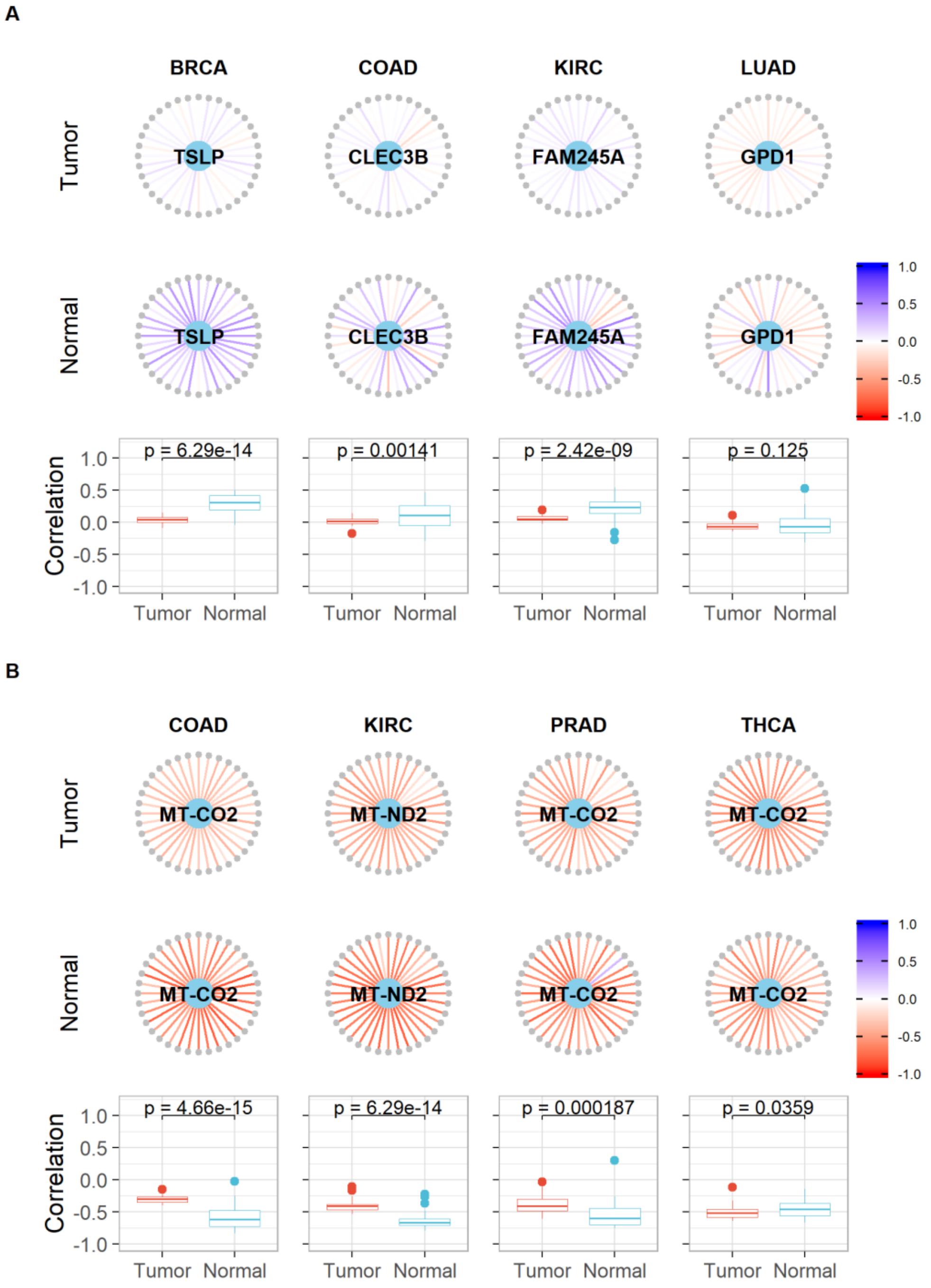

A) Shows differentially expressed core genes (light blue) and their interactions with other genes (grey) in the pathway while **B)** shows similar interactions with non-differentially expressed core genes. Correlations are indicated as edges ranging from red (-1) to blue (+1). Boxplots show the correlations indicated in the same pathways to highlight differences in distributions along with the p-value for the Kolmogorov-Smirnov test with the null hypothesis being that the two samples (correlations of Tumour and Normal tissues) were drawn from the same distribution.

Supplementary Figure S11 - Base excision repair (hsa03410) and some core gene interactions highlighted by SCOPE.

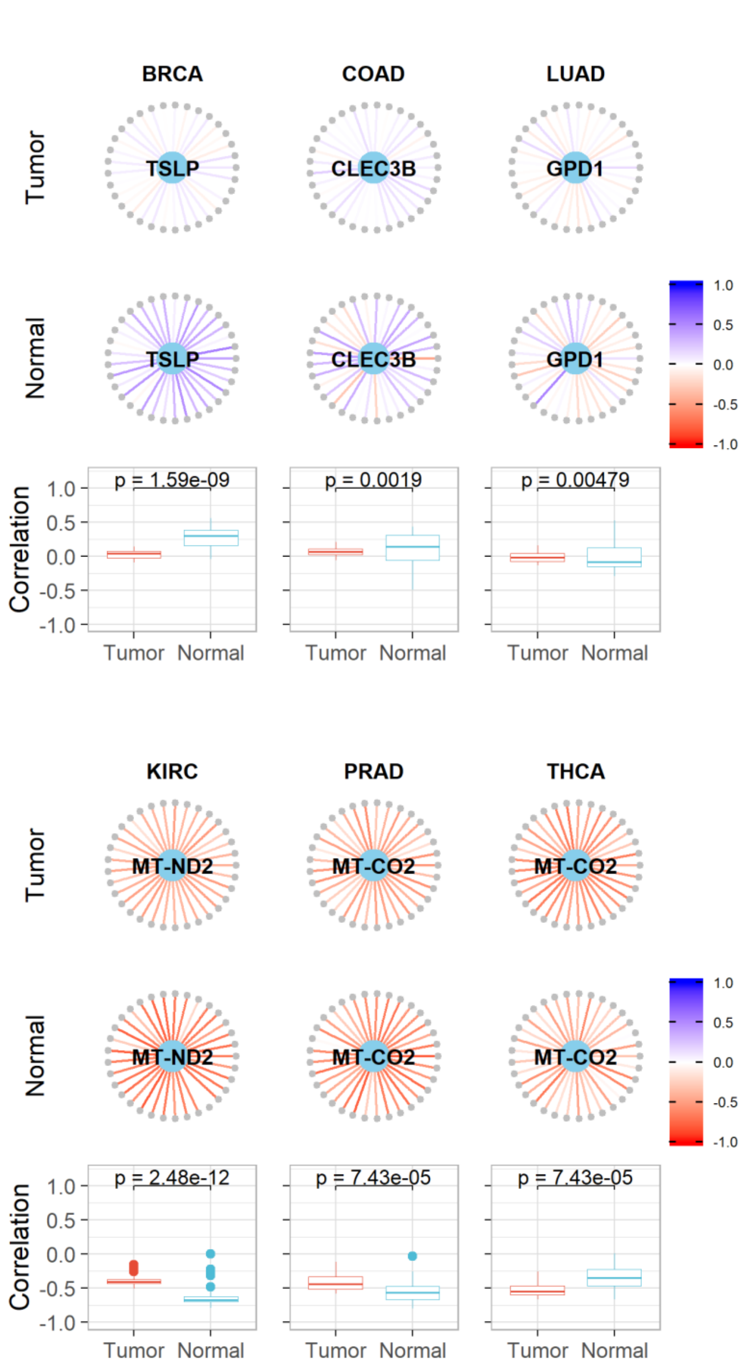

A) Shows differentially expressed core genes (light blue) and their interactions with other genes (grey) in the pathway while **B)** shows similar interactions with non-differentially expressed core genes. Correlations are indicated as edges ranging from red (-1) to blue (+1). Boxplots show the correlations indicated in the same pathways to highlight differences in distributions along with the p-value for the Kolmogorov-Smirnov test with the null hypothesis being that the two samples (correlations of Tumour and Normal tissues) were drawn from the same distribution.

Supplementary Figure S12 - Cell cycle (hsa04110) and some core gene interactions highlighted by SCOPE.

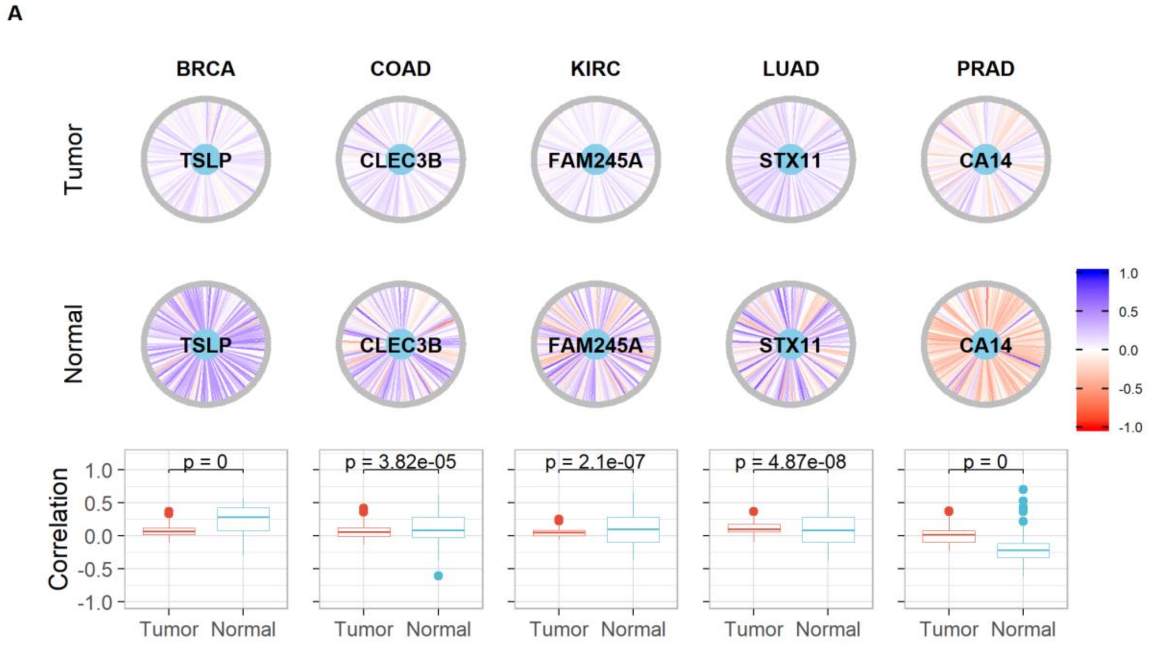

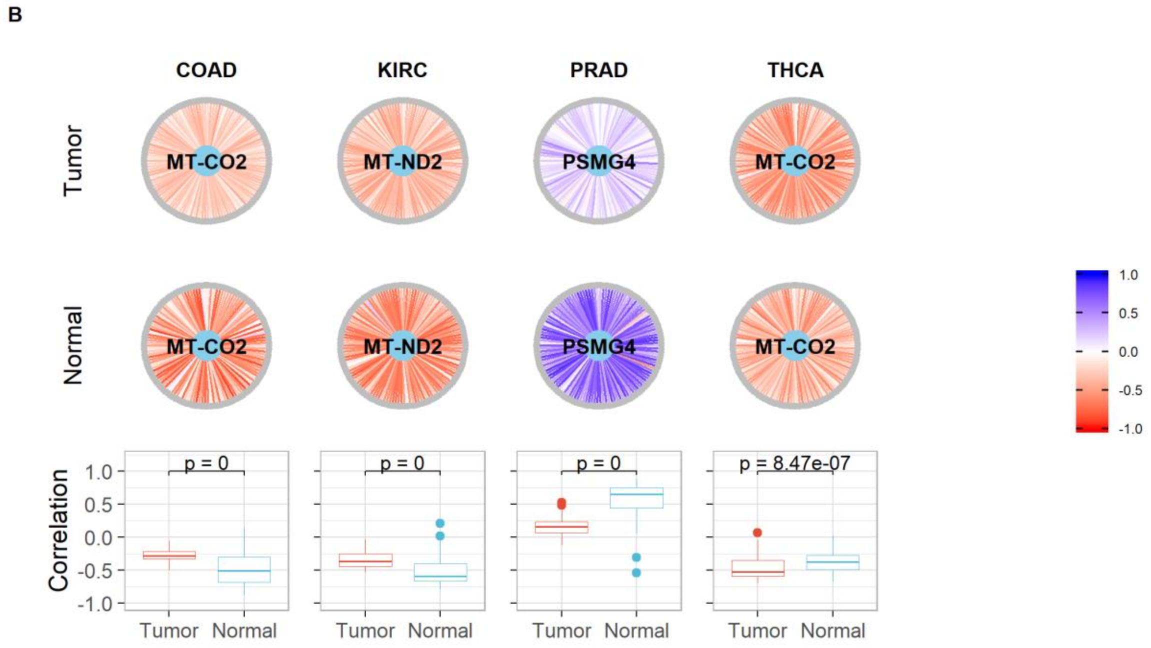

A) Shows differentially expressed core genes (light blue) and their interactions with other genes (grey) in the pathway while **B)** shows similar interactions with non-differentially expressed core genes. Correlations are indicated as edges ranging from red (-1) to blue (+1). Boxplots show the correlations indicated in the same pathways to highlight differences in distributions along with the p-value for the Kolmogorov-Smirnov test with the null hypothesis being that the two samples (correlations of Tumour and Normal tissues) were drawn from the same distribution.

Supplementary Figure S13 - Homologous recombination (hsa03440) and some core gene interactions highlighted by SCOPE.

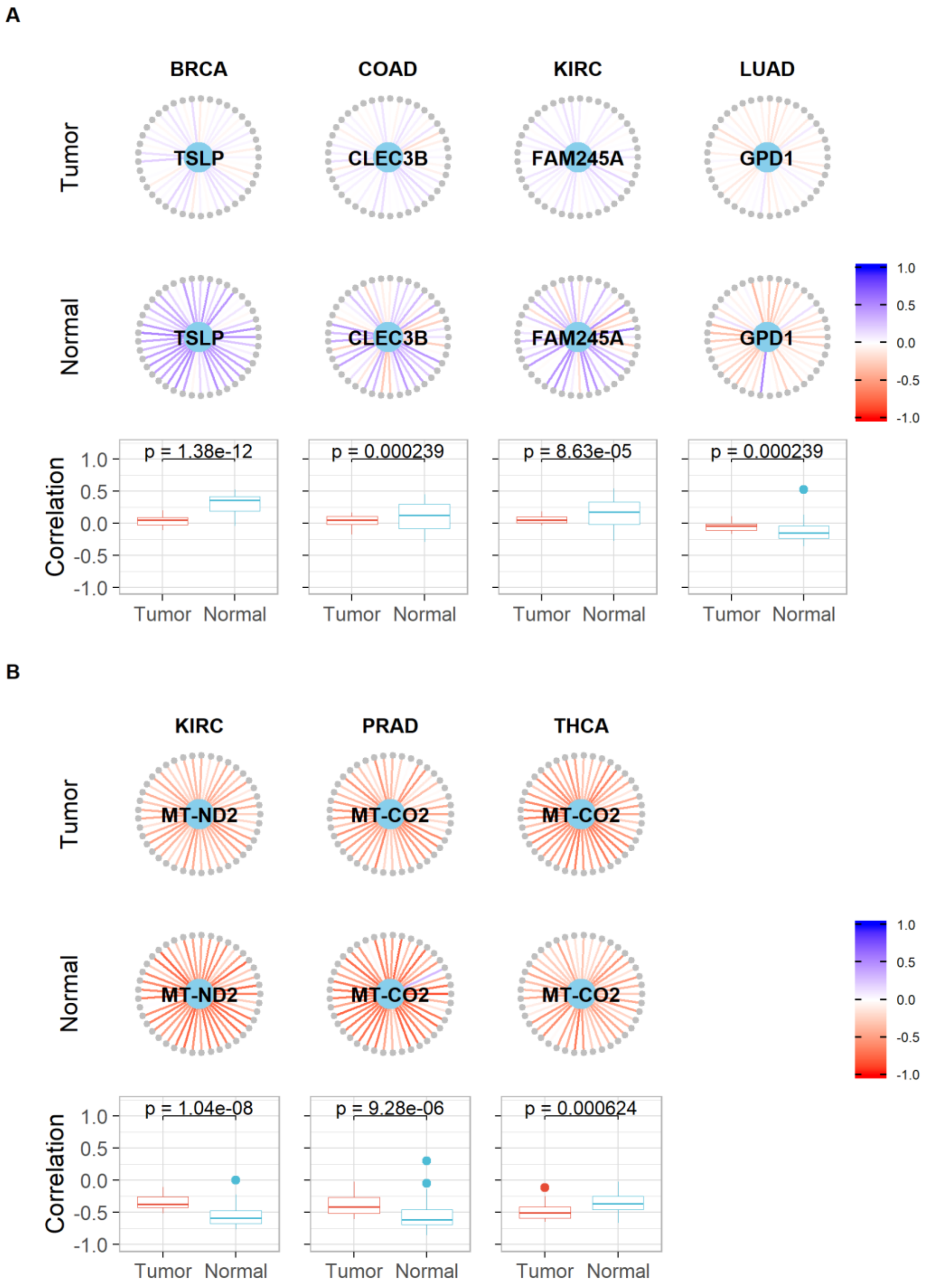

A) Shows differentially expressed core genes (light blue) and their interactions with other genes (grey) in the pathway while **B)** shows similar interactions with non-differentially expressed core genes. Correlations are indicated as edges ranging from red (-1) to blue (+1). Boxplots show the correlations indicated in the same pathways to highlight differences in distributions along with the p-value for the Kolmogorov-Smirnov test with the null hypothesis being that the two samples (correlations of Tumour and Normal tissues) were drawn from the same distribution.

Supplementary Figure S14 - p53 signaling pathway (hsa04115) and some core gene interactions highlighted by SCOPE.

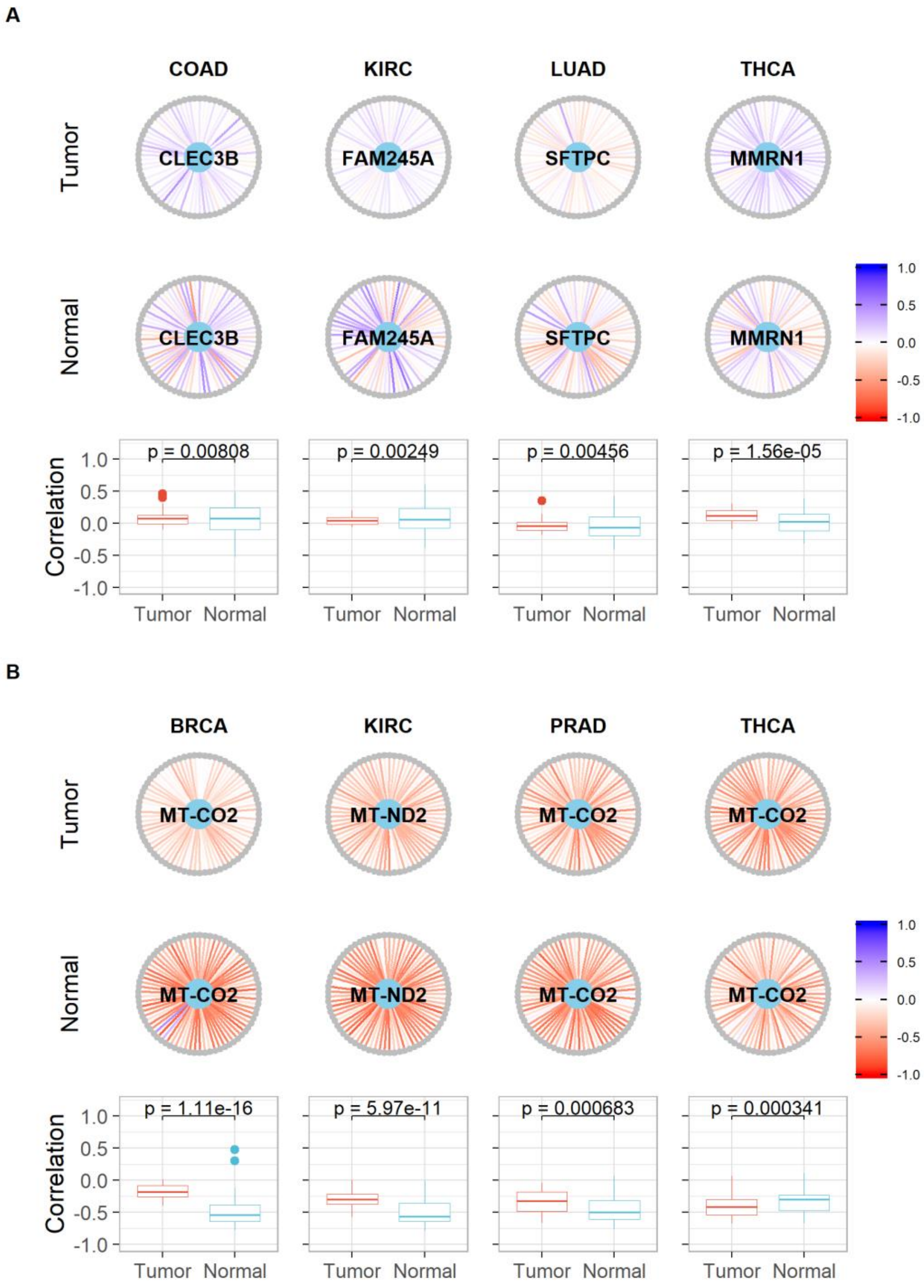

A) Shows differentially expressed core genes (light blue) and their interactions with other genes (grey) in the pathway while **B)** shows similar interactions with non-differentially expressed core genes. Correlations are indicated as edges ranging from red (-1) to blue (+1). Boxplots show the correlations indicated in the same pathways to highlight differences in distributions along with the p-value for the Kolmogorov-Smirnov test with the null hypothesis being that the two samples (correlations of Tumour and Normal tissues) were drawn from the same distribution.

Supplementary Figure S15 – Network plots of cancer specific pathways.

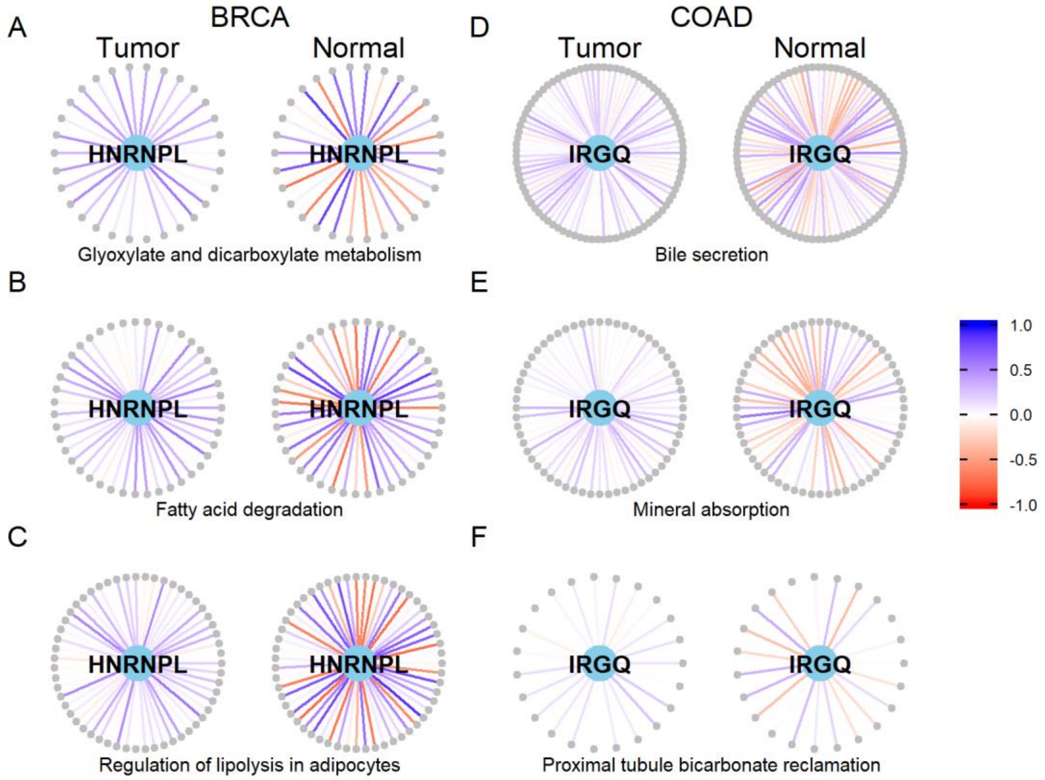

Core genes are indicated in light blue and other genes in the pathway, in grey. Correlations are indicated as edges ranging from red (-1) to blue (+1).

Supplementary Figure S16 – Survival curves of *CD63* over-expressed and under-expressed patients in all cancers.

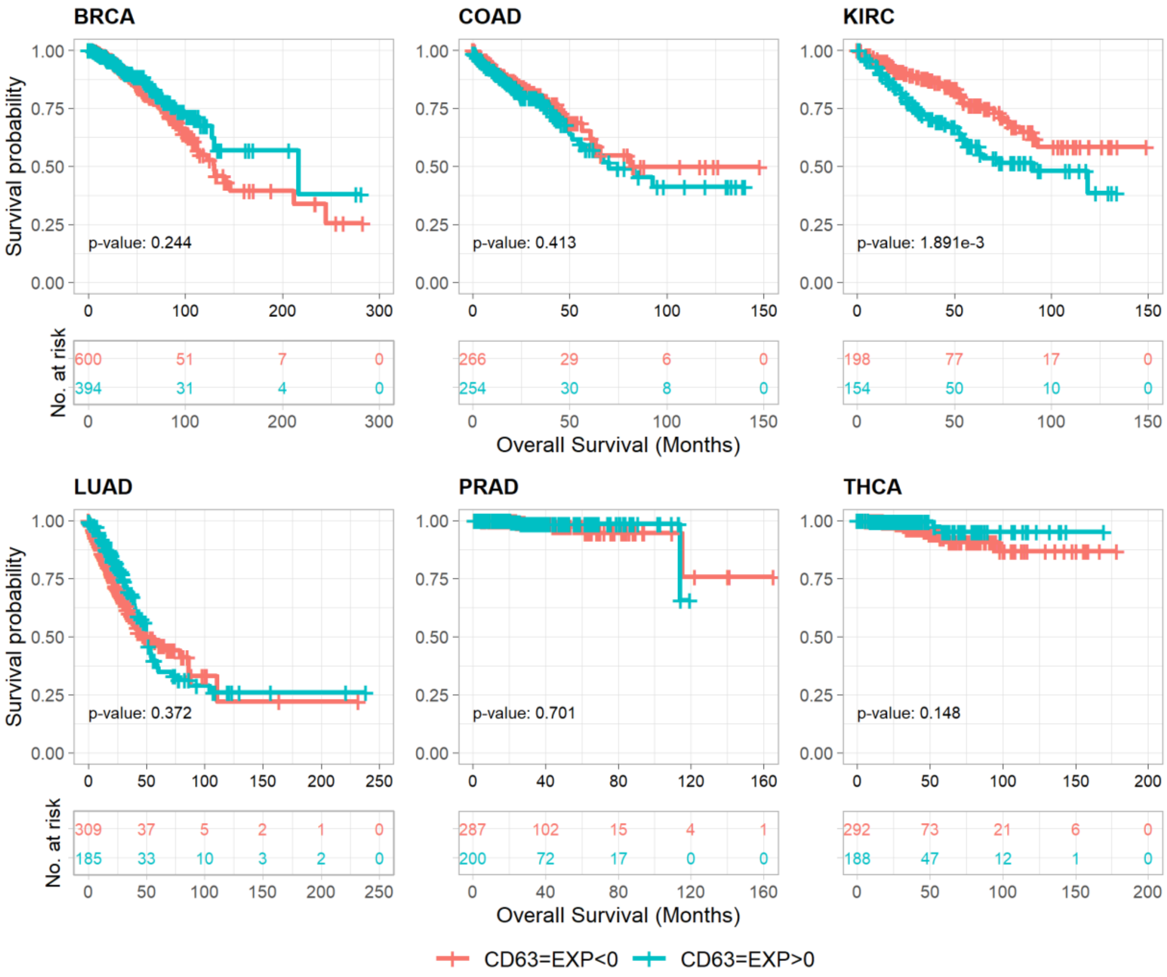

Log-rank tests indicate a significant difference in survival probabilities only for KIRC with respect to expression levels of *CD63*. (EXP < 0 indicates samples in which the expression level of the gene (*CD63*) is lower than the arithmetic mean of the expression levels of the gene across all samples; while EXP > 0 indicates higher than mean expression levels.)

Supplementary Figure S17 - PI3K-AKT-mTOR signaling pathway is highly mutated in BRCA.

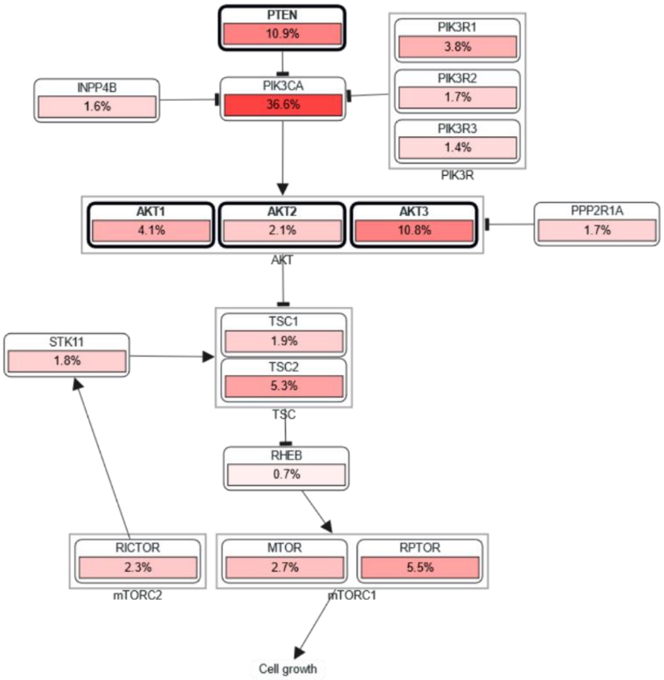

Pathway diagram obtained from cBioPortal.

Supplementary Table S1 – Predictive metrics of identifying causal genes in simulations using 670 samples of GTEx Data

Phenotype Model	Core Gene Correlations	Variance explained by phenotype	Model	TPR	FPR	TNR	FNR
**Linear**	**High**	**0.7**	**Adaptive Elastic Net**	0.277	0.126	0.874	0.723
			**Randomized LASSO**	0.051	0.001	0.999	0.949
			**SCOPE**	0.099	0.002	0.998	0.901
		**0.8**	**Adaptive Elastic Net**	0.307	0.151	0.849	0.693
			**Randomized LASSO**	0.074	0.001	0.999	0.926
			**SCOPE**	0.144	0.003	0.997	0.856
		**0.9**	**Adaptive Elastic Net**	0.360	0.184	0.816	0.640
			**Randomized LASSO**	0.097	0.002	0.998	0.903
			**SCOPE**	0.190	0.004	0.996	0.810
	**Low**	**0.7**	**Adaptive Elastic Net**	0.415	0.079	0.921	0.585
			**Randomized LASSO**	0.457	0.001	0.999	0.543
			**SCOPE**	0.418	0.001	0.999	0.582
		**0.8**	**Adaptive Elastic Net**	0.454	0.119	0.881	0.546
			**Randomized LASSO**	0.525	0.001	0.999	0.475
			**SCOPE**	0.525	0.002	0.998	0.475
		**0.9**	**Adaptive Elastic Net**	0.490	0.190	0.810	0.510
			**Randomized LASSO**	0.600	0.001	0.999	0.400
			**SCOPE**	0.642	0.002	0.998	0.358
**Non-linear**	**High**	**0.7**	**Adaptive Elastic Net**	0.796	0.023	0.977	0.204
			**Randomized LASSO**	0.299	0.000	1.000	0.701
			**SCOPE**	0.401	0.000	1.000	0.599
		**0.8**	**Adaptive Elastic Net**	0.879	0.028	0.972	0.121
			**Randomized LASSO**	0.344	0.000	1.000	0.656
			**SCOPE**	0.482	0.000	1.000	0.518
		**0.9**	**Adaptive Elastic Net**	0.917	0.047	0.953	0.083
			**Randomized LASSO**	0.406	0.000	1.000	0.594
			**SCOPE**	0.588	0.001	0.999	0.412
	**Low**	**0.7**	**Adaptive Elastic Net**	0.914	0.037	0.963	0.086
			**Randomized LASSO**	0.682	0.000	1.000	0.318
			**SCOPE**	0.675	0.000	1.000	0.325
		**0.8**	**Adaptive Elastic Net**	0.933	0.043	0.957	0.067
			**Randomized LASSO**	0.759	0.000	1.000	0.241
			**SCOPE**	0.851	0.000	1.000	0.149
		**0.9**	**Adaptive Elastic Net**	0.955	0.050	0.950	0.045
			**Randomized LASSO**	0.835	0.000	1.000	0.165
			**SCOPE**	0.972	0.001	0.999	0.028

Mean values of True Positive Rate (TPR), False Positive Rate (FPR), True Negative Rate (TNR) and False Negative Rate (FNR) in identifying causal genes (both core and extra genes involved in simulating the phenotype) by each of the models tested, in linear and non-linear scenarios under different signal-to-noise ratios (phenotypic variance explained) and correlation structures relative to core genes.

Supplementary Table S2 – Predictive metrics of identifying core pathways in simulations using 670 samples of GTEx Data based on the top 10 pathways identified

Phenotype Model	Core Gene Correlations	Variance explained by phenotype	Model	TPR	FPR	TNR	FNR
Linear	High	0.7	Adaptive Elastic Net	0.306	0.024	0.976	0.694
			Randomized LASSO	0.056	0.013	0.987	0.944
			SCOPE	0.358	0.016	0.984	0.642
		0.8	Adaptive Elastic Net	0.332	0.024	0.976	0.668
			Randomized LASSO	0.073	0.016	0.984	0.927
			SCOPE	0.379	0.015	0.985	0.621
		0.9	Adaptive Elastic Net	0.372	0.023	0.977	0.628
			Randomized LASSO	0.069	0.019	0.981	0.931
			SCOPE	0.378	0.014	0.986	0.622
	Low	0.7	Adaptive Elastic Net	0.297	0.024	0.976	0.703
			Randomized LASSO	0.114	0.028	0.972	0.886
			SCOPE	0.332	0.020	0.980	0.668
		0.8	Adaptive Elastic Net	0.337	0.024	0.976	0.663
			Randomized LASSO	0.099	0.028	0.972	0.901
			SCOPE	0.325	0.020	0.980	0.675
		0.9	Adaptive Elastic Net	0.401	0.022	0.978	0.599
			Randomized LASSO	0.104	0.028	0.972	0.896
			SCOPE	0.328	0.019	0.981	0.672
Non-linear	High	0.7	Adaptive Elastic Net	0.180	0.026	0.974	0.820
			Randomized LASSO	0.064	0.021	0.979	0.936
			SCOPE	0.317	0.011	0.989	0.683
		0.8	Adaptive Elastic Net	0.203	0.026	0.974	0.797
			Randomized LASSO	0.070	0.022	0.978	0.930
			SCOPE	0.295	0.011	0.989	0.705
		0.9	Adaptive Elastic Net	0.245	0.025	0.975	0.755
			Randomized LASSO	0.078	0.024	0.976	0.922
			SCOPE	0.280	0.010	0.990	0.720
	Low	0.7	Adaptive Elastic Net	0.238	0.025	0.975	0.762
			Randomized LASSO	0.122	0.028	0.972	0.878
			SCOPE	0.318	0.017	0.983	0.682
		0.8	Adaptive Elastic Net	0.281	0.025	0.975	0.719
			Randomized LASSO	0.127	0.028	0.972	0.873
			SCOPE	0.308	0.017	0.983	0.692
		0.9	Adaptive Elastic Net	0.268	0.025	0.975	0.732
			Randomized LASSO	0.134	0.028	0.972	0.866
			SCOPE	0.300	0.017	0.983	0.700

Mean values of True Positive Rate (TPR), False Positive Rate (FPR), True Negative Rate (TNR) and False Negative Rate (FNR) in identifying core pathways (based on the top 10 pathways ranked by FDR) by each of the models tested, in linear and non-linear scenarios under different signal-to-noise ratios (phenotypic variance explained) and correlation structures relative to core genes.

Supplementary Table S3 – Predictive metrics of identifying core pathways in simulations using 670 samples of GTEx Data based on all pathways identified under FDR<0.05

Phenotype Model	Core Gene Correlations	Variance explained by phenotype	Model	TPR	FPR	TNR	FNR
Linear	High	0.7	Adaptive Elastic Net	0.495	0.217	0.783	0.505
			Randomized LASSO	0.004	0.002	0.998	0.996
			SCOPE	0.599	0.324	0.676	0.401
		0.8	Adaptive Elastic Net	0.556	0.251	0.749	0.444
			Randomized LASSO	0.005	0.003	0.997	0.995
			SCOPE	0.668	0.393	0.607	0.332
		0.9	Adaptive Elastic Net	0.600	0.301	0.699	0.400
			Randomized LASSO	0.004	0.004	0.996	0.996
			SCOPE	0.704	0.429	0.571	0.296
	Low	0.7	Adaptive Elastic Net	0.413	0.126	0.874	0.587
			Randomized LASSO	0.009	0.003	0.997	0.991
			SCOPE	0.626	0.313	0.687	0.374
		0.8	Adaptive Elastic Net	0.513	0.188	0.812	0.487
			Randomized LASSO	0.012	0.004	0.996	0.988
			SCOPE	0.650	0.326	0.674	0.350
		0.9	Adaptive Elastic Net	0.627	0.271	0.729	0.373
			Randomized LASSO	0.016	0.004	0.996	0.984
			SCOPE	0.670	0.363	0.637	0.330
Non-linear	High	0.7	Adaptive Elastic Net	0.180	0.065	0.935	0.820
			Randomized LASSO	0.005	0.006	0.994	0.995
			SCOPE	0.708	0.486	0.514	0.292
		0.8	Adaptive Elastic Net	0.238	0.081	0.919	0.762
			Randomized LASSO	0.009	0.009	0.991	0.991
			SCOPE	0.715	0.497	0.503	0.285
		0.9	Adaptive Elastic Net	0.310	0.116	0.884	0.690
			Randomized LASSO	0.010	0.010	0.990	0.990
			SCOPE	0.726	0.506	0.494	0.274
	Low	0.7	Adaptive Elastic Net	0.269	0.067	0.933	0.731
			Randomized LASSO	0.022	0.002	0.998	0.978
			SCOPE	0.678	0.409	0.591	0.322
		0.8	Adaptive Elastic Net	0.326	0.081	0.919	0.674
			Randomized LASSO	0.021	0.004	0.996	0.979
			SCOPE	0.720	0.458	0.542	0.280
		0.9	Adaptive Elastic Net	0.351	0.100	0.900	0.649
			Randomized LASSO	0.029	0.005	0.995	0.971
			SCOPE	0.748	0.477	0.523	0.252

Mean values of True Positive Rate (TPR), False Positive Rate (FPR), True Negative Rate (TNR) and False Negative Rate (FNR) in identifying core pathways (based on all pathways identified under FDR<0.05) by each of the models tested, in linear and non-linear scenarios under different signal-to-noise ratios (phenotypic variance explained) and correlation structures relative to core genes.

Supplementary Table S4 – Predictive metrics of identifying core genes in simulations using 670 samples of GTEx Data

Phenotype Model	Core Gene Correlations	Variance explained by phenotype	Model	TPR	FPR	TNR	FNR
Linear	High	0.7	Adaptive Elastic Net	0.268	0.126	0.874	0.732
			Randomized LASSO	0.012	0.001	0.999	0.988
			SCOPE	0.070	0.003	0.997	0.930
		0.8	Adaptive Elastic Net	0.299	0.151	0.849	0.701
			Randomized LASSO	0.021	0.002	0.998	0.979
			SCOPE	0.098	0.004	0.996	0.902
		0.9	Adaptive Elastic Net	0.351	0.184	0.816	0.649
			Randomized LASSO	0.027	0.003	0.997	0.973
			SCOPE	0.133	0.005	0.995	0.867
	Low	0.7	Adaptive Elastic Net	0.444	0.080	0.920	0.556
			Randomized LASSO	0.530	0.001	0.999	0.470
			SCOPE	0.467	0.002	0.998	0.533
		0.8	Adaptive Elastic Net	0.482	0.120	0.880	0.518
			Randomized LASSO	0.599	0.001	0.999	0.401
			SCOPE	0.582	0.003	0.997	0.418
		0.9	Adaptive Elastic Net	0.500	0.191	0.809	0.500
			Randomized LASSO	0.663	0.002	0.998	0.337
			SCOPE	0.695	0.004	0.996	0.305
Non-linear	High	0.7	Adaptive Elastic Net	0.821	0.026	0.974	0.179
			Randomized LASSO	0.238	0.002	0.998	0.762
			SCOPE	0.379	0.002	0.998	0.621
		0.8	Adaptive Elastic Net	0.908	0.030	0.970	0.092
			Randomized LASSO	0.286	0.002	0.998	0.714
			SCOPE	0.463	0.002	0.998	0.537
		0.9	Adaptive Elastic Net	0.930	0.050	0.950	0.070
			Randomized LASSO	0.331	0.003	0.997	0.669
			SCOPE	0.555	0.003	0.997	0.445
	Low	0.7	Adaptive Elastic Net	0.916	0.040	0.960	0.084
			Randomized LASSO	0.685	0.002	0.998	0.315
			SCOPE	0.665	0.002	0.998	0.335
		0.8	Adaptive Elastic Net	0.928	0.046	0.954	0.072
			Randomized LASSO	0.761	0.002	0.998	0.239
			SCOPE	0.848	0.003	0.997	0.152
		0.9	Adaptive Elastic Net	0.955	0.053	0.947	0.045
			Randomized LASSO	0.837	0.003	0.997	0.163
			SCOPE	0.971	0.004	0.996	0.029

Mean values of True Positive Rate (TPR), False Positive Rate (FPR), True Negative Rate (TNR) and False Negative Rate (FNR) in identifying core genes by each of the models tested, in linear and non-linear scenarios under different signal-to-noise ratios (phenotypic variance explained) and correlation structures relative to core genes.

Supplementary Table S5 – Predictive metrics of identifying causal genes in simulations using 500 samples of GTEx Data

Phenotype Model	Core Gene Correlations	Variance explained by phenotype	Model	TPR	FPR	TNR	FNR
Linear	High	0.7	Adaptive Elastic Net	0.259	0.095	0.905	0.741
			Randomized LASSO	0.048	0.001	0.999	0.952
			SCOPE	0.087	0.002	0.998	0.913
		0.8	Adaptive Elastic Net	0.345	0.146	0.854	0.655
			Randomized LASSO	0.073	0.001	0.999	0.927
			SCOPE	0.118	0.003	0.997	0.882
		0.9	Adaptive Elastic Net	0.358	0.170	0.830	0.642
			Randomized LASSO	0.093	0.001	0.999	0.907
			SCOPE	0.166	0.004	0.996	0.834
	Low	0.7	Adaptive Elastic Net	0.388	0.063	0.937	0.612
			Randomized LASSO	0.458	0.001	0.999	0.542
			SCOPE	0.417	0.001	0.999	0.583
		0.8	Adaptive Elastic Net	0.421	0.077	0.923	0.579
			Randomized LASSO	0.538	0.001	0.999	0.462
			SCOPE	0.538	0.002	0.998	0.462
		0.9	Adaptive Elastic Net	0.472	0.120	0.880	0.528
			Randomized LASSO	0.606	0.001	0.999	0.394
			SCOPE	0.647	0.002	0.998	0.353
Non-linear	High	0.7	Adaptive Elastic Net	0.729	0.021	0.979	0.271
			Randomized LASSO	0.244	0.001	0.999	0.756
			SCOPE	0.310	0.000	1.000	0.690
		0.8	Adaptive Elastic Net	0.804	0.025	0.975	0.196
			Randomized LASSO	0.280	0.001	0.999	0.720
			SCOPE	0.403	0.000	1.000	0.597
		0.9	Adaptive Elastic Net	0.877	0.039	0.961	0.123
			Randomized LASSO	0.319	0.001	0.999	0.681
			SCOPE	0.492	0.000	1.000	0.508
	Low	0.7	Adaptive Elastic Net	0.861	0.036	0.964	0.139
			Randomized LASSO	0.612	0.000	1.000	0.388
			SCOPE	0.458	0.000	1.000	0.542
		0.8	Adaptive Elastic Net	0.921	0.045	0.955	0.079
			Randomized LASSO	0.677	0.000	1.000	0.323
			SCOPE	0.669	0.000	1.000	0.331
		0.9	Adaptive Elastic Net	0.944	0.056	0.944	0.056
			Randomized LASSO	0.755	0.000	1.000	0.245
			SCOPE	0.879	0.001	0.999	0.121

Mean values of True Positive Rate (TPR), False Positive Rate (FPR), True Negative Rate (TNR) and False Negative Rate (FNR) in identifying causal genes (both core and extra genes involved in simulating the phenotype) by each of the models tested, in linear and non-linear scenarios under different signal-to-noise ratios (phenotypic variance explained) and correlation structures relative to core genes.

Supplementary Table S6 – Predictive metrics of identifying core pathways in simulations using 500 samples of GTEx Data based on the top 10 pathways identified

Phenotype Model	Core Gene Correlations	Variance explained by phenotype	Model	TPR	FPR	TNR	FNR
Linear	High	0.7	Adaptive Elastic Net	0.328	0.025	0.975	0.672
			Randomized LASSO	0.059	0.012	0.988	0.941
			SCOPE	0.369	0.017	0.983	0.631
		0.8	Adaptive Elastic Net	0.382	0.024	0.976	0.618
			Randomized LASSO	0.074	0.014	0.986	0.926
			SCOPE	0.419	0.017	0.983	0.581
		0.9	Adaptive Elastic Net	0.396	0.024	0.976	0.604
			Randomized LASSO	0.091	0.017	0.983	0.909
			SCOPE	0.430	0.016	0.984	0.570
	Low	0.7	Adaptive Elastic Net	0.287	0.025	0.975	0.713
			Randomized LASSO	0.144	0.025	0.975	0.856
			SCOPE	0.342	0.020	0.980	0.658
		0.8	Adaptive Elastic Net	0.315	0.025	0.975	0.685
			Randomized LASSO	0.174	0.025	0.975	0.826
			SCOPE	0.368	0.020	0.980	0.632
		0.9	Adaptive Elastic Net	0.359	0.024	0.976	0.641
			Randomized LASSO	0.163	0.026	0.974	0.837
			SCOPE	0.370	0.020	0.980	0.630
Non-linear	High	0.7	Adaptive Elastic Net	0.198	0.026	0.974	0.802
			Randomized LASSO	0.073	0.018	0.982	0.927
			SCOPE	0.359	0.011	0.989	0.641
		0.8	Adaptive Elastic Net	0.217	0.025	0.975	0.783
			Randomized LASSO	0.085	0.019	0.981	0.915
			SCOPE	0.366	0.011	0.989	0.634
		0.9	Adaptive Elastic Net	0.247	0.026	0.974	0.753
			Randomized LASSO	0.083	0.022	0.978	0.917
			SCOPE	0.356	0.011	0.989	0.644
	Low	0.7	Adaptive Elastic Net	0.255	0.025	0.975	0.745
			Randomized LASSO	0.150	0.025	0.975	0.850
			SCOPE	0.300	0.016	0.984	0.700
		0.8	Adaptive Elastic Net	0.285	0.025	0.975	0.715
			Randomized LASSO	0.173	0.026	0.974	0.827
			SCOPE	0.370	0.017	0.983	0.630
		0.9	Adaptive Elastic Net	0.313	0.025	0.975	0.687
			Randomized LASSO	0.158	0.026	0.974	0.842
			SCOPE	0.367	0.017	0.983	0.633

Mean values of True Positive Rate (TPR), False Positive Rate (FPR), True Negative Rate (TNR) and False Negative Rate (FNR) in identifying core pathways (based on the top 10 pathways ranked by FDR) by each of the models tested, in linear and non-linear scenarios under different signal-to-noise ratios (phenotypic variance explained) and correlation structures relative to core genes.

Supplementary Table S7 – Predictive metrics of identifying core pathways in simulations using 500 samples of GTEx Data based on all pathways identified under FDR<0.05

Phenotype Model	Core Gene Correlations	Variance explained by phenotype	Model	TPR	FPR	TNR	FNR
Linear	High	0.7	Adaptive Elastic Net	0.514	0.156	0.844	0.486
			Randomized LASSO	0.002	0.001	0.999	0.998
			SCOPE	0.664	0.261	0.739	0.336
		0.8	Adaptive Elastic Net	0.636	0.219	0.781	0.364
			Randomized LASSO	0.002	0.002	0.998	0.998
			SCOPE	0.763	0.329	0.671	0.237
		0.9	Adaptive Elastic Net	0.715	0.253	0.747	0.285
			Randomized LASSO	0.002	0.002	0.998	0.998
			SCOPE	0.817	0.371	0.629	0.183
	Low	0.7	Adaptive Elastic Net	0.280	0.064	0.936	0.720
			Randomized LASSO	0.001	0.004	0.996	0.999
			SCOPE	0.582	0.253	0.747	0.418
		0.8	Adaptive Elastic Net	0.368	0.095	0.905	0.632
			Randomized LASSO	0.004	0.005	0.995	0.996
			SCOPE	0.657	0.288	0.712	0.343
		0.9	Adaptive Elastic Net	0.488	0.151	0.849	0.512
			Randomized LASSO	0.001	0.006	0.994	0.999
			SCOPE	0.667	0.319	0.681	0.333
Non-linear	High	0.7	Adaptive Elastic Net	0.157	0.037	0.963	0.843
			Randomized LASSO	0.004	0.002	0.998	0.996
			SCOPE	0.780	0.398	0.602	0.220
		0.8	Adaptive Elastic Net	0.184	0.045	0.955	0.816
			Randomized LASSO	0.002	0.003	0.997	0.998
			SCOPE	0.821	0.436	0.564	0.179
		0.9	Adaptive Elastic Net	0.305	0.074	0.926	0.695
			Randomized LASSO	0.005	0.003	0.997	0.995
			SCOPE	0.856	0.465	0.535	0.144
	Low	0.7	Adaptive Elastic Net	0.228	0.054	0.946	0.772
			Randomized LASSO	0.001	0.004	0.996	0.999
			SCOPE	0.530	0.230	0.770	0.470
		0.8	Adaptive Elastic Net	0.317	0.072	0.928	0.683
			Randomized LASSO	0.001	0.003	0.997	0.999
			SCOPE	0.692	0.325	0.675	0.308
		0.9	Adaptive Elastic Net	0.349	0.088	0.912	0.651
			Randomized LASSO	0.002	0.005	0.995	0.998
			SCOPE	0.730	0.368	0.632	0.270

Mean values of True Positive Rate (TPR), False Positive Rate (FPR), True Negative Rate (TNR) and False Negative Rate (FNR) in identifying core pathways (based on all pathways identified under FDR<0.05) by each of the models tested, in linear and non-linear scenarios under different signal-to-noise ratios (phenotypic variance explained) and correlation structures relative to core genes.

Supplementary Table S8 – Predictive metrics of identifying core genes in simulations using 500 samples of GTEx Data

Phenotype Model	Core Gene Correlations	Variance explained by phenotype	Model	TPR	FPR	TNR	FNR
Linear	High	0.7	Adaptive Elastic Net	0.255	0.095	0.905	0.745
Linear	High	0.7	Randomized LASSO	0.006	0.002	0.998	0.994
Linear	High	0.7	SCOPE	0.056	0.002	0.998	0.944
Linear	High	0.8	Adaptive Elastic Net	0.340	0.146	0.854	0.660
Linear	High	0.8	Randomized LASSO	0.018	0.002	0.998	0.982
Linear	High	0.8	SCOPE	0.076	0.004	0.996	0.924
Linear	High	0.9	Adaptive Elastic Net	0.355	0.171	0.829	0.645
Linear	High	0.9	Randomized LASSO	0.022	0.003	0.997	0.978
Linear	High	0.9	SCOPE	0.106	0.005	0.995	0.894
Linear	Low	0.7	Adaptive Elastic Net	0.418	0.064	0.936	0.582
Linear	Low	0.7	Randomized LASSO	0.536	0.001	0.999	0.464
Linear	Low	0.7	SCOPE	0.474	0.002	0.998	0.526
Linear	Low	0.8	Adaptive Elastic Net	0.435	0.078	0.922	0.565
Linear	Low	0.8	Randomized LASSO	0.607	0.002	0.998	0.393
Linear	Low	0.8	SCOPE	0.593	0.003	0.997	0.407
Linear	Low	0.9	Adaptive Elastic Net	0.498	0.121	0.879	0.502
Linear	Low	0.9	Randomized LASSO	0.684	0.002	0.998	0.316
Linear	Low	0.9	SCOPE	0.712	0.004	0.996	0.288
Non-linear	High	0.7	Adaptive Elastic Net	0.758	0.024	0.976	0.242
Non-linear	High	0.7	Randomized LASSO	0.197	0.002	0.998	0.803
Non-linear	High	0.7	SCOPE	0.312	0.001	0.999	0.688
Non-linear	High	0.8	Adaptive Elastic Net	0.832	0.028	0.972	0.168
Non-linear	High	0.8	Randomized LASSO	0.231	0.002	0.998	0.769
Non-linear	High	0.8	SCOPE	0.390	0.002	0.998	0.610
Non-linear	High	0.9	Adaptive Elastic Net	0.899	0.042	0.958	0.101
Non-linear	High	0.9	Randomized LASSO	0.250	0.003	0.997	0.750
Non-linear	High	0.9	SCOPE	0.465	0.003	0.997	0.535
Non-linear	Low	0.7	Adaptive Elastic Net	0.855	0.039	0.961	0.145
Non-linear	Low	0.7	Randomized LASSO	0.618	0.002	0.998	0.382
Non-linear	Low	0.7	SCOPE	0.446	0.002	0.998	0.554
Non-linear	Low	0.8	Adaptive Elastic Net	0.919	0.049	0.951	0.081
Non-linear	Low	0.8	Randomized LASSO	0.682	0.002	0.998	0.318
Non-linear	Low	0.8	SCOPE	0.659	0.003	0.997	0.341
Non-linear	Low	0.9	Adaptive Elastic Net	0.943	0.059	0.941	0.057
Non-linear	Low	0.9	Randomized LASSO	0.761	0.003	0.997	0.239
Non-linear	Low	0.9	SCOPE	0.874	0.004	0.996	0.126

Mean values of True Positive Rate (TPR), False Positive Rate (FPR), True Negative Rate (TNR) and False Negative Rate (FNR) in identifying core genes by each of the models tested, in linear and non-linear scenarios under different signal-to-noise ratios (phenotypic variance explained) and correlation structures relative to core genes.

Supplementary Table S9 – Predictive metrics of identifying causal genes in simulations using 250 samples of GTEx Data

Phenotype Model	Core Gene Correlations	Variance explained by phenotype	Model	TPR	FPR	TNR	FNR
Linear	High	0.7	Adaptive Elastic Net	0.159	0.040	0.960	0.841
			Randomized LASSO	0.016	0.001	0.999	0.984
			SCOPE	0.027	0.001	0.999	0.973
		0.8	Adaptive Elastic Net	0.187	0.054	0.946	0.813
			Randomized LASSO	0.025	0.001	0.999	0.975
			SCOPE	0.049	0.001	0.999	0.951
		0.9	Adaptive Elastic Net	0.229	0.087	0.913	0.771
			Randomized LASSO	0.035	0.001	0.999	0.965
			SCOPE	0.068	0.002	0.998	0.932
	Low	0.7	Adaptive Elastic Net	0.280	0.038	0.962	0.720
			Randomized LASSO	0.267	0.000	1.000	0.733
			SCOPE	0.192	0.001	0.999	0.808
		0.8	Adaptive Elastic Net	0.332	0.051	0.949	0.668
			Randomized LASSO	0.348	0.000	1.000	0.652
			SCOPE	0.307	0.001	0.999	0.693
		0.9	Adaptive Elastic Net	0.368	0.065	0.935	0.632
			Randomized LASSO	0.425	0.001	0.999	0.575
			SCOPE	0.419	0.001	0.999	0.581
Non-linear	High	0.7	Adaptive Elastic Net	0.513	0.010	0.990	0.487
			Randomized LASSO	0.076	0.000	1.000	0.924
			SCOPE	0.160	0.000	1.000	0.840
		0.8	Adaptive Elastic Net	0.587	0.014	0.986	0.413
			Randomized LASSO	0.105	0.000	1.000	0.895
			SCOPE	0.212	0.000	1.000	0.788
		0.9	Adaptive Elastic Net	0.694	0.021	0.979	0.306
			Randomized LASSO	0.151	0.000	1.000	0.849
			SCOPE	0.275	0.000	1.000	0.725
	Low	0.7	Adaptive Elastic Net	0.436	0.013	0.987	0.564
			Randomized LASSO	0.344	0.000	1.000	0.656
			SCOPE	0.124	0.000	1.000	0.876
		0.8	Adaptive Elastic Net	0.629	0.021	0.979	0.371
			Randomized LASSO	0.421	0.000	1.000	0.579
			SCOPE	0.210	0.000	1.000	0.790
		0.9	Adaptive Elastic Net	0.776	0.028	0.972	0.224
			Randomized LASSO	0.511	0.000	1.000	0.489
			SCOPE	0.368	0.000	1.000	0.632

Mean values of True Positive Rate (TPR), False Positive Rate (FPR), True Negative Rate (TNR) and False Negative Rate (FNR) in identifying causal genes (both core and extra genes involved in simulating the phenotype) by each of the models tested, in linear and non-linear scenarios under different signal-to-noise ratios (phenotypic variance explained) and correlation structures relative to core genes.

Supplementary Table S10 – Predictive metrics of identifying core pathways in simulations using 250 samples of GTEx Data based on the top 10 pathways identified

Phenotype Model	Core Gene Correlations	Variance explained by phenotype	Model	TPR	FPR	TNR	FNR
Linear	High	0.7	**Adaptive Elastic Net**	0.174	0.026	0.974	0.826
			**Randomized LASSO**	0.028	0.007	0.993	0.972
			**SCOPE**	0.130	0.014	0.986	0.870
		0.8	**Adaptive Elastic Net**	0.180	0.026	0.974	0.820
			**Randomized LASSO**	0.030	0.008	0.992	0.970
			**SCOPE**	0.157	0.019	0.981	0.843
		0.9	**Adaptive Elastic Net**	0.202	0.026	0.974	0.798
			**Randomized LASSO**	0.055	0.011	0.989	0.945
			**SCOPE**	0.193	0.020	0.980	0.807
	Low	0.7	**Adaptive Elastic Net**	0.203	0.026	0.974	0.797
			**Randomized LASSO**	0.193	0.022	0.978	0.807
			**SCOPE**	0.145	0.017	0.983	0.855
		0.8	**Adaptive Elastic Net**	0.237	0.025	0.975	0.763
			**Randomized LASSO**	0.198	0.025	0.975	0.802
			**SCOPE**	0.189	0.022	0.978	0.811
		0.9	**Adaptive Elastic Net**	0.238	0.025	0.975	0.762
			**Randomized LASSO**	0.257	0.025	0.975	0.743
			**SCOPE**	0.205	0.023	0.977	0.795
Non-linear	High	0.7	**Adaptive Elastic Net**	0.078	0.024	0.976	0.922
			**Randomized LASSO**	0.037	0.009	0.991	0.963
			**SCOPE**	0.111	0.011	0.989	0.889
		0.8	**Adaptive Elastic Net**	0.094	0.025	0.975	0.906
			**Randomized LASSO**	0.041	0.012	0.988	0.959
			**SCOPE**	0.139	0.013	0.987	0.861
		0.9	**Adaptive Elastic Net**	0.117	0.027	0.973	0.883
			**Randomized LASSO**	0.052	0.015	0.985	0.948
			**SCOPE**	0.153	0.014	0.986	0.847
	Low	0.7	**Adaptive Elastic Net**	0.158	0.018	0.982	0.842
			**Randomized LASSO**	0.182	0.021	0.979	0.818
			**SCOPE**	0.058	0.006	0.994	0.942
		0.8	**Adaptive Elastic Net**	0.248	0.021	0.979	0.752
			**Randomized LASSO**	0.200	0.022	0.978	0.800
			**SCOPE**	0.092	0.010	0.990	0.908
		0.9	**Adaptive Elastic Net**	0.267	0.024	0.976	0.733
			**Randomized LASSO**	0.232	0.024	0.976	0.768
			**SCOPE**	0.126	0.015	0.985	0.874

Mean values of True Positive Rate (TPR), False Positive Rate (FPR), True Negative Rate (TNR) and False Negative Rate (FNR) in identifying core pathways (based on the top 10 pathways ranked by FDR) by each of the models tested, in linear and non-linear scenarios under different signal-to-noise ratios (phenotypic variance explained) and correlation structures relative to core genes.

Supplementary Table S11 – Predictive metrics of identifying core pathways in simulations using 250 samples of GTEx Data based on all pathways identified under FDR<0.05

Phenotype Model	Core Gene Correlations	Variance explained by phenotype	Model	TPR	FPR	TNR	FNR
Linear	High	0.7	Adaptive Elastic Net	0.177	0.062	0.938	0.823
			Randomized LASSO	0.000	0.000	1.000	1.000
			SCOPE	0.272	0.129	0.871	0.728
		0.8	Adaptive Elastic Net	0.241	0.098	0.902	0.759
			Randomized LASSO	0.001	0.000	1.000	0.999
			SCOPE	0.375	0.199	0.801	0.625
		0.9	Adaptive Elastic Net	0.324	0.130	0.870	0.676
			Randomized LASSO	0.003	0.000	1.000	0.997
			SCOPE	0.461	0.254	0.746	0.539
	Low	0.7	Adaptive Elastic Net	0.192	0.043	0.957	0.808
			Randomized LASSO	0.002	0.000	1.000	0.998
			SCOPE	0.385	0.165	0.835	0.615
		0.8	Adaptive Elastic Net	0.283	0.057	0.943	0.717
			Randomized LASSO	0.003	0.000	1.000	0.997
			SCOPE	0.562	0.244	0.756	0.438
		0.9	Adaptive Elastic Net	0.343	0.083	0.917	0.657
			Randomized LASSO	0.007	0.000	1.000	0.993
			SCOPE	0.621	0.278	0.722	0.379
Non-linear	High	0.7	Adaptive Elastic Net	0.058	0.029	0.971	0.942
			Randomized LASSO	0.002	0.001	0.999	0.998
			SCOPE	0.378	0.253	0.747	0.622
		0.8	Adaptive Elastic Net	0.071	0.034	0.966	0.929
			Randomized LASSO	0.005	0.002	0.998	0.995
			SCOPE	0.471	0.306	0.694	0.529
		0.9	Adaptive Elastic Net	0.123	0.050	0.950	0.877
			Randomized LASSO	0.002	0.001	0.999	0.998
			SCOPE	0.557	0.363	0.637	0.443
	Low	0.7	Adaptive Elastic Net	0.071	0.013	0.987	0.929
			Randomized LASSO	0.004	0.000	1.000	0.996
			SCOPE	0.160	0.071	0.929	0.840
		0.8	Adaptive Elastic Net	0.152	0.023	0.977	0.848
			Randomized LASSO	0.003	0.000	1.000	0.997
			SCOPE	0.261	0.125	0.875	0.739
		0.9	Adaptive Elastic Net	0.197	0.031	0.969	0.803
			Randomized LASSO	0.009	0.000	1.000	0.991
			SCOPE	0.442	0.214	0.786	0.558

Mean values of True Positive Rate (TPR), False Positive Rate (FPR), True Negative Rate (TNR) and False Negative Rate (FNR) in identifying core pathways (based on all pathways identified under FDR<0.05) by each of the models tested, in linear and non-linear scenarios under different signal-to-noise ratios (phenotypic variance explained) and correlation structures relative to core genes.

Supplementary Table S12 – Predictive metrics of identifying core genes in simulations using 250 samples of GTEx Data

Phenotype Model	Core Gene Correlations	Variance explained by phenotype	Model	TPR	FPR	TNR	FNR
**Linear**	**High**	**0.7**	**Adaptive Elastic Net**	0.153	0.040	0.960	0.847
			**Randomized LASSO**	0.001	0.001	0.999	0.999
			**SCOPE**	0.018	0.001	0.999	0.982
		**0.8**	**Adaptive Elastic Net**	0.178	0.055	0.945	0.822
			**Randomized LASSO**	0.001	0.001	0.999	0.999
			**SCOPE**	0.028	0.002	0.998	0.972
		**0.9**	**Adaptive Elastic Net**	0.214	0.087	0.913	0.786
			**Randomized LASSO**	0.002	0.001	0.999	0.998
			**SCOPE**	0.035	0.003	0.997	0.965
	**Low**	**0.7**	**Adaptive Elastic Net**	0.305	0.038	0.962	0.695
			**Randomized LASSO**	0.325	0.001	0.999	0.675
			**SCOPE**	0.225	0.001	0.999	0.775
		**0.8**	**Adaptive Elastic Net**	0.366	0.052	0.948	0.634
			**Randomized LASSO**	0.421	0.001	0.999	0.579
			**SCOPE**	0.355	0.001	0.999	0.645
		**0.9**	**Adaptive Elastic Net**	0.396	0.065	0.935	0.604
			**Randomized LASSO**	0.504	0.001	0.999	0.496
			**SCOPE**	0.477	0.002	0.998	0.523
**Non-linear**	**High**	**0.7**	**Adaptive Elastic Net**	0.545	0.011	0.989	0.455
			**Randomized LASSO**	0.050	0.001	0.999	0.950
			**SCOPE**	0.163	0.000	1.000	0.837
		**0.8**	**Adaptive Elastic Net**	0.622	0.016	0.984	0.378
			**Randomized LASSO**	0.070	0.001	0.999	0.930
			**SCOPE**	0.208	0.001	0.999	0.792
		**0.9**	**Adaptive Elastic Net**	0.719	0.023	0.977	0.281
			**Randomized LASSO**	0.098	0.002	0.998	0.902
			**SCOPE**	0.255	0.001	0.999	0.745
	**Low**	**0.7**	**Adaptive Elastic Net**	0.428	0.014	0.986	0.572
			**Randomized LASSO**	0.352	0.001	0.999	0.648
			**SCOPE**	0.114	0.000	1.000	0.886
		**0.8**	**Adaptive Elastic Net**	0.622	0.023	0.977	0.378
			**Randomized LASSO**	0.431	0.001	0.999	0.569
			**SCOPE**	0.197	0.001	0.999	0.803
		**0.9**	**Adaptive Elastic Net**	0.770	0.031	0.969	0.230
			**Randomized LASSO**	0.519	0.002	0.998	0.481
			**SCOPE**	0.362	0.001	0.999	0.638

Mean values of True Positive Rate (TPR), False Positive Rate (FPR), True Negative Rate (TNR) and False Negative Rate (FNR) in identifying core genes by each of the models tested, in linear and non-linear scenarios under different signal-to-noise ratios (phenotypic variance explained) and correlation structures relative to core genes.

Supplementary Table S13 – Core genes identified for each of the 6 selected cancers of the TCGA database.

BRCA		COAD		KIRC		LUAD		PRAD		THCA	
**Gene**	θ_{BRCA}	**Gene**	θ_{COAD}	**Gene**	θ_{KIRC}	**Gene**	θ_{LUAD}	**Gene**	θ_{PRAD}	**Gene**	θ_{THCA}
HNRNPL	1.000	***SCARA5***	1.000	***FAM245A***	0.973	***EXOSC1***	0.999	***MT-CO2***	1.000	***RPS27L***	1.000
PAMR1	1.000	***TRIM27***	1.000	***MT-ND2***	0.958	***SFTPC***	0.998	***APOBEC3C***	1.000	***MT-CO2***	1.000
VEGFD	1.000	***ANAPC7***	0.999	***CORO7***	0.913	***GMPPA***	0.938	***ALDH1L1-AS2***	0.828	***CTSA***	0.984
OST4	1.000	***IRGQ***	0.873	***CD63***	0.810	***PMM2***	0.932	***CA14***	0.773	***CCL21***	0.965
MT-CO2	0.996	***TMIGD1***	0.871	***STX4***	0.753	***STX11***	0.874	***PSMG4***	0.755	***LINC01834***	0.879
TSLP	0.993	***CLEC3B***	0.814	*MPV17*	0.736	***GPD1***	0.785	*AAAS*	0.734	***DPT***	0.876
CD300LG	0.991	***MT-CO2***	0.791	*ANKRD39*	0.726	***GYPE***	0.779	*GPR89B*	0.719	***AL034374.1***	0.763
OXTR	0.988	*BMP3*	0.747	*TCF21*	0.680	***FABP4***	0.771	*POLR2H*	0.601	***MMRN1***	0.761
ARF1	0.961	*CA2*	0.716	*GGT6*	0.664	*LINC01996*	0.743	*GAR1*	0.599	***RYR2***	0.756
RNF149	0.955	*MT-ND4*	0.632	*DTX2*	0.644	*C1orf131*	0.688	*RAB17*	0.536	*RELN*	0.683

The θ value of each gene denotes the proportion of models in which the gene was selected. Since this was based on 1,000 independent LASSO runs, i.e., a value of 0.779 for *GYPE* gene in LUAD indicates that *GYPE* was selected by 779 of the LASSO models run for the LUAD dataset. Core genes selected based on the θ_{thr}s are indicated in bold.

Supplementary Table S14 – Stability of the SCOPE-Stabilized LASSO step.

BRCA		COAD		KIRC		LUAD		PRAD		THCA	
**Gene**	**Prop**	**Gene**	**Prop**	**Gene**	**Prop**	**Gene**	**Prop**	**Gene**	**Prop**	**Gene**	**Prop**
HNRNPL	1.00	*IRGQ*	1.00	*CD63*	1.00	*STX11*	1.00	*CA14*	1.00	*CTSA*	1.00
ARF1	1.00	*SCARA5*	1.00	*MT-ND2*	1.00	*PMM2*	1.00	*MT-CO2*	1.00	*CCL21*	1.00
TSLP	1.00	*TMIGD1*	1.00	*FAM245A*	1.00	*GMPPA*	1.00	*APOBEC3C*	1.00	*DPT*	1.00
PAMR1	1.00	*ANAPC7*	1.00	*CORO7*	1.00	*GPD1*	1.00	*ALDH1L1-AS2*	1.00	*RPS27L*	1.00
CD300LG	1.00	*MT-CO2*	1.00	*STX4*	0.69	*SFTPC*	1.00	*AAAS*	0.53	*MT-CO2*	1.00
RNF149	1.00	*TRIM27*	1.00	<u>*ANKRD39*</u>	<u>0.48</u>	*EXOSC1*	1.00	*PSMG4*	0.37	*LINC01834*	1.00
VEGFD	1.00	*CLEC3B*	0.99	<u>*MPV17*</u>	<u>0.30</u>	*GYPE*	1.00	<u>*GPR89B*</u>	<u>0.01</u>	*MMRN1*	0.90
OXTR	1.00	<u>*CA2*</u>	<u>0.54</u>			<u>*LINC01996*</u>	<u>0.87</u>			*AL034374.1*	0.90
MT-CO2	1.00	<u>*BMP3*</u>	<u>0.23</u>			*FABP4*	0.83			*RYR2*	0.37
OST4	1.00										

Among 100 runs, the proportion of runs selecting the gene as a core gene is listed in the right half of each column. "Prop" value of each gene denotes the proportion of SCOPE-Stabilized LASSO models that the gene was selected in. Since 100 SCOPE-Stabilized LASSO runs were used to obtain the results shown in this table, i.e., a value of 0.83 for FABP4 in LUAD indicates this gene was selected in 83 of the SCOPE-Stabilized LASSO runs for LUAD. Underlined genes are genes that were not selected in the SCOPE-Stabilized LASSO run used for the analysis in this paper.

Supplementary Table S15 – Within cancer pathway overlap across Core Gene Networks.

KEGG GeneSet	Pathway Name	BRCA (/10)	COAD (/7)	KIRC (/5)	LUAD (/8)	PRAD (/5)	THCA (/9)	Total (/44)
hsa04110	Cell cycle	5	4	2	5	4	2	22
hsa03460	Fanconi anemia pathway	5	4	1	5	2	1	18
hsa00190	Oxidative phosphorylation	6	2	4	2	1	2	17
hsa03030	DNA replication	5	4	2	4	1	1	17
hsa05012	Parkinson disease	6	2	3	2	1	3	17
hsa00240	Pyrimidine metabolism	5	4	2	3	1	1	16
hsa00510	N-Glycan biosynthesis	4	2	1	5	1	3	16
hsa03050	Proteasome	6	4	1	2	1	2	16
hsa03410	Base excision repair	5	3	1	4	1	2	16
hsa04141	Protein processing in endoplasmic reticulum	5	1	1	4	2	3	16
hsa04714	Thermogenesis	6	2	2	2	2	2	16
hsa03040	Spliceosome	5	4	1	3	1	1	15
hsa03440	Homologous recombination	5	3	2	3	1	1	15
hsa03420	Nucleotide excision repair	4	4	1	1	2	2	14
hsa04115	p53 signaling pathway	1	3	2	1	1	6	14
hsa03013	RNA transport	4	4	1	2	1	1	13

The number of CGNs that uncovered each pathway as enriched in each cancer is given in the table. This table shows the top 10 pathways sorted by the number of CGNs the pathway was uncovered in.

Supplementary Table S16 – Within cancer pathway overlaps across modules using DiffCoEx.

**Pathway Name**	**BRCA (/3)**	**COAD (/17)**	**KIRC (/10)**	**LUAD (/52)**	**PRAD (/7)**	**THCA (/33)**	**Total (/122)**
Cytokine-cytokine receptor interaction	1	1	0	3	1	3	9
AGE-RAGE signaling pathway in diabetic complications	1	1	1	3	1	1	8
Cell cycle	1	2	0	2	1	2	8
Epstein-Barr virus infection	0	2	0	3	1	2	8
Human T-cell leukemia virus 1 infection	0	1	1	3	1	2	8
Osteoclast differentiation	1	1	1	2	1	2	8
Spliceosome	1	1	0	2	2	2	8
Autophagy	0	2	0	2	1	2	7
Cellular senescence	1	1	1	2	1	1	7
Chagas disease (American trypanosomiasis)	1	1	0	3	1	1	7

The number of modules that uncovered each pathway as enriched in each cancer is given in the table. This table shows the top 10 pathways sorted by the number of modules the pathway was uncovered in.

Supplementary Table S17 – Distribution of proportion of genes identified by each SCOPE, DiffCoEx and DE across all pathways in each cancer along with their Pathway Overlap Scores (POS).

π_{cancer} values denote the proportion of genes contained in CGNs (for SCOPE), contained in differentially co-expressed modules (for DiffCoEx) and contained in the list of differentially expressed genes (for DE) out of the total number of genes in each pathway the proportion of genes. POS score is the summation of this value over all cancers for each method. Higher values indicate higher overlap of pathway across cancers.

(Refer Supplementary Excel file)

Supplementary Table S18 – Table of core genes and secondary genes involved in each of the pathways shown in Figures S10-S14.

Pearson Correlation Coefficients of gene pairs are included for each cancer and tissue type. (Refer Supplementary Excel File)

Supplementary Table S19 – Correlation patterns of Secondary Genes in CGN of *PSMG4* in PRAD.

**Core Gene**	**Secondary Gene**	$\mathbf{Corr_{tumo}}$	**p-value ($\mathbf{Corr_{tumor}}$)**	$\mathbf{Corr_{norm}}$	**p-value ($\mathbf{Corr_{normal}}$)**	$\boldsymbol{\lvert Corr_{tumor} - Corr_{norm}}$
PSMG4	*ACSL4*	-0.165	4.0E-04	0.618	1.7E-06	0.783
PSMG4	*MAP1LC3B*	0.040	4.0E-01	0.767	8.4E-11	0.727
PSMG4	*ATG5*	0.033	4.7E-01	0.753	2.9E-10	0.719
PSMG4	*PRNP*	-0.100	3.2E-02	0.602	3.7E-06	0.702
PSMG4	*NCOA4*	-0.007	8.8E-01	0.687	3.6E-08	0.694
PSMG4	*PCBP1*	0.099	3.3E-02	0.776	3.8E-11	0.676
PSMG4	*LPCAT3*	0.015	7.5E-01	0.688	3.5E-08	0.673
PSMG4	*VDAC3*	0.151	1.2E-03	0.821	2.8E-13	0.670
PSMG4	*FTH1*	0.158	6.9E-04	0.812	8.8E-13	0.653
PSMG4	*SLC39A14*	0.007	8.9E-01	0.652	2.9E-07	0.645
PSMG4	*SLC40A1*	-0.086	6.5E-02	0.541	5.1E-05	0.627
PSMG4	*SLC11A2*	0.107	2.2E-02	0.730	1.7E-09	0.623
PSMG4	*PCBP2*	0.176	1.6E-04	0.776	3.5E-11	0.601
PSMG4	*VDAC2*	0.239	2.3E-07	0.824	1.9E-13	0.585
PSMG4	*GCLC*	0.053	2.6E-01	0.636	7.1E-07	0.583
PSMG4	*ACSL6*	-0.033	4.8E-01	-0.575	1.3E-05	0.542

The absolute differences in correlations reveals significantly switched correlations in cancer and normal tissues in genes involved in the Ferroptosis pathway.

Supplementary Table S20 – Detailed breakdown of all genes identified to be enriched in pathways of Core Gene Networks (CGN).

Pearson correlation coefficients across normal, tumor and all samples as well as the differential co-expression between normal and tumor are included. Critical values obtained for differential co-expression as well as results of the limma-voom pipeline for differential expression for both core genes and the secondary genes are included.
(Refer Supplementary Excel File)

Supplementary Table S21 – Breakdown of sample sizes across the 6 different cancers chosen from the TCGA database.

TCGA Name	Cancer	Primary Tumor	Normal Tissue
BRCA	Breast Invasive Carcinoma	1,041	111
KIRC	Kidney Renal Clear Cell Carcinoma	480	70
LUAD	Lung Adenocarcinoma	483	54
COAD	Colon Adenocarcinoma	387	37
PRAD	Prostate Adenocarcinoma	458	50
THCA	Thyroid Carcinoma	444	53
